# Jasmonic acid biosynthetic genes *TgLOX4* and *TgLOX5* are involved in daughter bulb development in tulip (*Tulipa gesneriana*)

**DOI:** 10.1093/hr/uhac006

**Published:** 2022-02-11

**Authors:** Qi Sun, Bei Zhang, Chaolong Yang, Weiliang Wang, Lin Xiang, Yanping Wang, Zhulong Chan

**Affiliations:** Key Laboratory of Horticultural Plant Biology, Ministry of Education, College of Horticulture and Forestry Sciences, Huazhong Agricultural University, Wuhan, 430070, China; Key Laboratory of Horticultural Plant Biology, Ministry of Education, College of Horticulture and Forestry Sciences, Huazhong Agricultural University, Wuhan, 430070, China; Key Laboratory of Horticultural Plant Biology, Ministry of Education, College of Horticulture and Forestry Sciences, Huazhong Agricultural University, Wuhan, 430070, China; Key Laboratory of Horticultural Plant Biology, Ministry of Education, College of Horticulture and Forestry Sciences, Huazhong Agricultural University, Wuhan, 430070, China; Key Laboratory of Horticultural Plant Biology, Ministry of Education, College of Horticulture and Forestry Sciences, Huazhong Agricultural University, Wuhan, 430070, China; Key Laboratory of Horticultural Plant Biology, Ministry of Education, College of Horticulture and Forestry Sciences, Huazhong Agricultural University, Wuhan, 430070, China; Key Laboratory of Horticultural Plant Biology, Ministry of Education, College of Horticulture and Forestry Sciences, Huazhong Agricultural University, Wuhan, 430070, China

## Abstract

Tulip bulbs are modified underground stems that originate from axillary meristems of mother bulb scales. Hormones, including jasmonic acids (JAs), play key roles in the regulation of tulip bulb development. Here, we compared variations in daughter bulb development through transcriptomic profiling analysis and characterized the functions of JA biosynthesis-related genes during daughter bulb enlargement. The results showed that tulip cultivars exhibited contrasting bulb size variations. Transcriptomic analyses revealed that genes involved in plant hormones and development, including the two lipoxygenase genes *TgLOX4* and *TgLOX5*, showed significant changes in expression following tulip bulb growth. Ectopic overexpression of *TgLOX4* and *TgLOX5* in *Arabidopsis* enhanced endogenous JA content, improved plant growth, and increased lateral root numbers. Silencing of these two genes in tulip repressed the growth of daughter bulbs. Furthermore, exogenous JA treatment promoted tulip bulb growth, whereas the JA biosynthesis inhibitor sodium diethyldithiocarbamate (DIECA) inhibited this process. This study offers supporting evidence for the involvement of tulip *TgLOX4* and *TgLOX5* in the regulation of daughter bulb growth and development.

## Introduction

Tulip (*Tulipa gesneriana* L.) is an ornamental bulbous plant that is widely used for landscaping and cut flowers [1, 2]. The *Tulipa* genus is distributed in the Mediterranean, Central Asia, Europe and Northern Africa [3–5]. Tulips have a long juvenile phase for up to 3–7 years [6]. Therefore, tulips are mainly propagated vegetatively through bulb proliferation. Seed propagation is used only for the breeding of new cultivars because of the long adult vegetative phase and high heterozygosity [7]. Tulip bulbs are modified underground stems that consist of a brown, dry tunic outside, several layers of modified leaves called scales, and an abnormally short stem called the basal plate. In tulip, floral meristem initiation and differentiation occur inside the expanded bulbs during the summer season [8, 9]. A mature flowering tulip bulb, referred to as a mother bulb, contains one apical meristem and six axillary meristems [8, 10]. The aboveground stems, leaves, and floral organs of tulip plants are developed from the apical meristem, and the axillary meristems expand as bulblets (daughter bulbs) [11].

Plant hormones are key regulators of plant growth and development [12, 13]. Jasmonates, represented by jasmonic acid (JA) and its volatile methyl ester (methyl jasmonate, MeJA), are pivotal plant growth regulators that control plant stress responses, flowering, and development [13, 14]. The first isolated JA compound was MeJA, which was initially identified as an odorant in *Jasminum grandiflorum* flowers [15]. The signaling perception pathway of JA has been well characterized. Binding of JA to the F-box protein CORONATINE INSENSITIVE1 (COI1) leads to the degradation of JASMONATE ZIM (JAZ) and the activation of the basic-helix–loop–helix (bHLH) transcription factor *MYC2*. The biosynthetic pathway of JA from α-linolenic acid through the octadecanoid pathway was established in the 1980s. α-Linolenic acid is subsequently converted into the intermediate 12-oxo-phytodienoic acid (OPDA) by a lipoxygenase (LOX), an allene oxide synthase (AOS), and an allene oxide cyclase (AOC) [16, 17].

LOXs (EC 1.13.1.13) are non-heme, nonsulfur oxidoreductases that are widely present in living organisms, including mammals, plants, fishes, mosses, bacteria, yeast, fungi, corals, algae, and mushrooms [18]. Plant LOXs are classified into two major subfamilies, 9-LOXs and 13-LOXs. 9-LOXs are predominantly involved in plant defense responses against various pathogens, whereas 13-LOXs play key roles in the biosynthesis of JA and volatiles [19]. *Arabidopsis* 13-LOXs, especially LOX2, LOX3, LOX4, and LOX6, can produce JA precursors in leaves [20, 21]. In tomato, 14 *LOX* gene family members were identified and exhibited differential associations with growth, development, and fruit ripening [22]. In potato, JA has been shown to be associated with the induction of radial cell expansion in tubers and tuber buds [23, 24]. The potato *LOX1* gene is highly expressed in newly formed tubers. Suppression of LOX1 class activity resulted in reduced tuber yields and disruption of normal tuber morphology [19]. Legume *LOX* mRNAs and proteins were detected in nodules, mainly in the developing stage, but their expression and activity levels decreased in nodules of complete size [25]. All these results indicate that the modulation of *LOX* genes and changes in JA content contribute to the promotion of plant growth and development.

**Figure 1:**
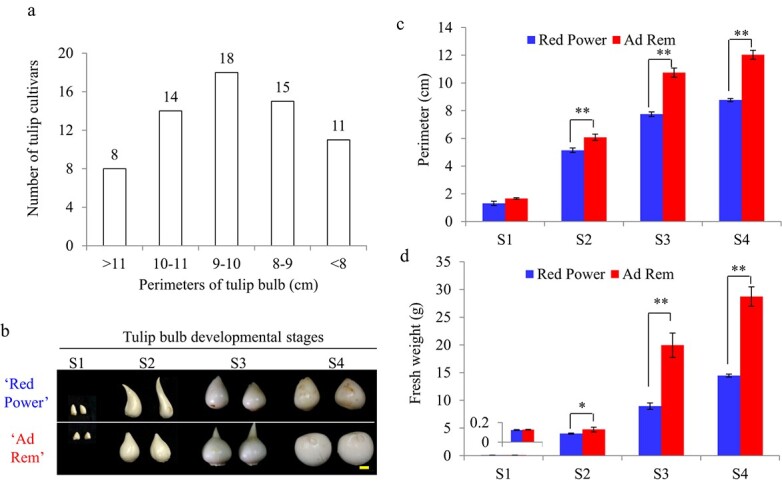
Bulb growth rates of two tulip cultivars. (a) Perimeter size distribution of daughter bulbs from 66 tulip cultivars. (b) Bulb photos at four developmental stages. (c) Perimeters of bulbs at four developmental stages. (d) Fresh weights of bulbs at four developmental stages. Student’s *t*-test was used to analyze statistical significance (^*^P < 0.05, ^**^P < 0.01). S1, bulblets inside the mother bulbs after dormancy release in late January; S2, bulblets from tulip plants with green buds 4–5 cm in length in early March; S3, bulbs one week after full bloom in middle (“Red Power”) and late (“Ad Rem”) March; and S4, bulbs from plants that were senescent in April. Bar = 1 cm.

In tulip, MeJA treatment induced fatty acid and sterol concentrations in stems [26]. Exogenous application of polyamines (PAs) and MeJA significantly improved tulip bulb formation [27]. To date, the mechanism by which JAs regulate tulip bulb growth remains to be investigated. The functions of JA pathway–related genes involved in this process are elusive. In this study, we aimed to determine the effects of exogenous JAs and a JA biosynthesis inhibitor on tulip bulb formation and swelling. The functions of tulip *LOX* genes in the JA pathway were also dissected through ectopic expression in *Arabidopsis* plants and repressed expression in tulip by virus-induced gene silencing (VIGS). The results provide new clues for understanding the mechanisms of tulip bulb development.

## Results

### Variation in daughter bulb size among tulip cultivars

In this study, natural variations in tulip bulb size were first investigated. The daughter bulb perimeters of 66 tulip cultivars varied from 6.7 cm to 13.4 cm (Fig. S1). The perimeters of daughter bulbs of the majority of cultivars were 8.0–11.0 cm when plants were cultivated in Wuhan, China (113°41′–115°05′E, 29°58′–31°22′N) (Fig. 1a). Based on this preliminary investigation, the two tulip cultivars “Ad Rem” and “Red Power” with red flowers and contrasting daughter bulb sizes were selected for further study (Fig. S1). We observed that there was significant enlargement of daughter bulbs from stage 1 to stage 2 in both cultivars. “Ad Rem” exhibited slightly smaller daughter bulbs compared with “Red Power” at stage 1 before planting, but “Ad Rem” bulbs were significantly larger than those of “Red Power” at stage 4 (Fig. 1b, 1c). Consequently, the fresh weight of “Ad Rem” bulbs was significantly higher than that of “Red Power” bulbs from S2 to S4 (Fig. 1d).

### Transcriptomic changes during tulip daughter bulb development

**Figure 2:**
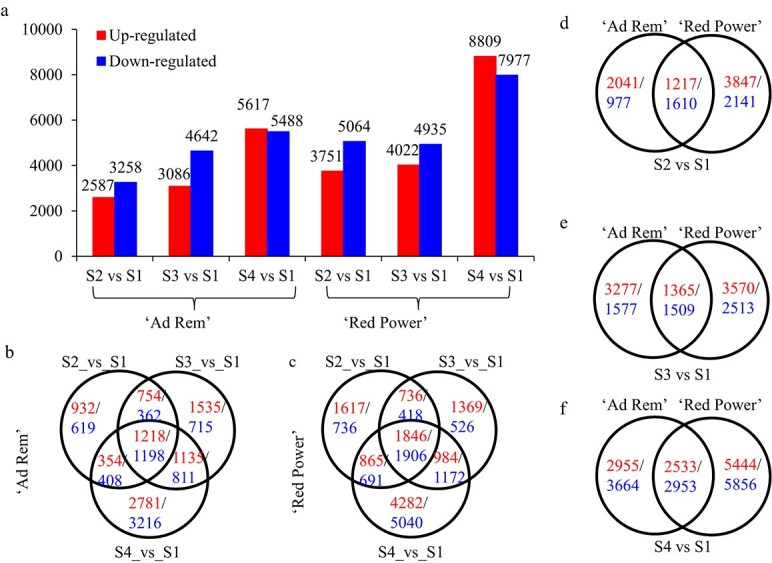
Transcriptomic analysis of two tulip cultivars at four developmental stages. (a) Numbers of genes whose expression changed at different stages in two cultivars. The original gene expression data are provided in Table S2. (b,c) Overlapping analysis of changed genes at four developmental stages in two cultivars. (d,e) Overlapped genes between two tulip cultivars in S2_vsS1, S3_vsS1, and S4_vsS1. The original gene expression data are provided in Table S2. S1, bulblets inside the mother bulbs after dormancy release in late January; S2, bulblets from tulip plants with green buds 4–5 cm in length in early March; S3, bulbs one week after full bloom in middle (“Red Power”) and late (“Ad Rem”) March; and S4, bulbs from plants that were senescent in April.

Daughter bulbs of the two tulip cultivars at four developmental stages were collected for RNA sequencing analysis. In cultivar “Ad Rem”, a total of 5845, 7728, and 11 105 unigenes showed significant expression changes in S2_vs_S1, S3_vs_S1, and S4_vs_S1, respectively. In cultivar “Red Power”, the numbers of changed unigenes were 8815, 8957, and 16 786 for S2_vs_S1, S3_vs_S1, and S4_vs_S1, respectively (Fig. 2a; Table S1). Overlapping analysis showed that 2416 (1218 upregulated and 1198 downregulated) and 3752 (1846 upregulated and 1906 downregulated) unigenes were commonly regulated in three stages in “Ad Rem” and “Red Power”, respectively (Fig. 2b, 2c). In total, 23.9%, 20.8%, and 23.4% of unigenes were co-regulated in the two cultivars in S2_vs_S1, S3_vs_S1, and S4_vs_S1, respectively (Fig. 2d–f).

GO term enrichment analysis indicated that GO terms including regulation of cell proliferation, meiotic cell cycle, cell wall, macromolecule, and metabolic process were over-represented only in “Ad Rem” (Fig. S2a). Other GO terms related to development (cell cycle, cell proliferation, cell division, cell growth, cell differentiation, etc.) and hormone pathways (hormone transport, regulation of hormone levels, and response to hormone stimulus) were enriched in both tulip cultivars (Fig. S2b). These results indicated that hormone- and development-related pathways were extensively changed during tulip bulb growth.

Pathway enrichment analysis was performed using MapMAN software. The results indicated that twelve pathways were overrepresented in S2 to S4 relative to S1 in the two tulip cultivars: fermentation, major CHO metabolism, biodegradation of xenobiotics, amino acid metabolism, minor CHO metabolism, TCA/org transformation, cell wall, transport, secondary metabolism, lipid metabolism, cell, and hormone metabolism (Table S2). Another eight pathways were enriched in most of the four developmental stages in both cultivars: gluconeogenesis/glyoxylate cycle, oxidative phosphorylation, glycolysis, polyamine metabolism, N-metabolism, nucleotide metabolism, redox, and C1-metabolism (Table S2).

### Changes in the JA pathway during bulb development

**Figure 3:**
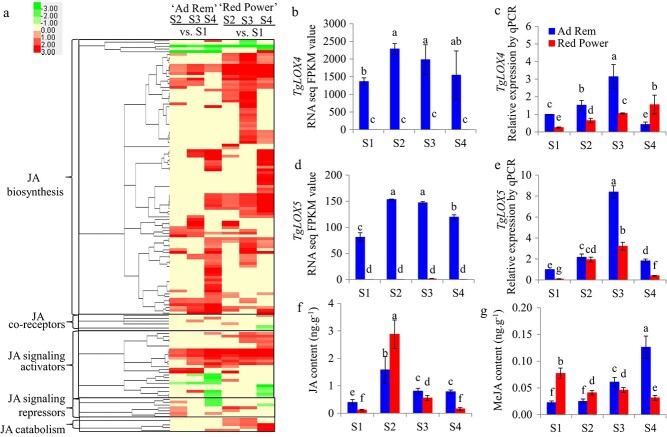
Expression level changes in JA pathway–related genes in two tulip cultivars at four developmental stages. (a) Hierarchical clustering analysis of genes involved in JA biosynthesis and signal transduction pathways performed with the Cluster 3.0 software package. The resulting tree figure was visualized using Java TreeView. (b,d) Expression of *TgLOX4* and *TgLOX5* measured by RNA sequencing. (c,e) Expression of *TgLOX4* and *TgLOX5* measured by real-time qPCR. (f) JA content. Information on all unigenes is provided in Table S3. Letters indicate statistically significant differences determined by Duncan’s multiple range test at the *P* ≤ 0.05 level. S1, bulblets inside the mother bulbs after dormancy release in late January; S2, bulblets from tulip plants with green buds 4–5 cm in length in early March; S3, bulbs one week after full bloom in middle (“Red Power”) and late (“Ad Rem”) March; and S4, bulbs from plants that were senescent in April. The qPCR primer sequences for *TgLOX4* and *TgLOX5* are provided in Fig. S3B and S4B, respectively.

Transcriptomic data showed that 142 unigenes involved in the JA pathway exhibited significant changes in expression level following tulip bulb growth in both cultivars, including 100 unigenes encoding JA biosynthesis enzymes, 6 unigenes encoding JA co-receptors, 24 unigenes encoding JA signaling activators, 7 unigenes encoding JA signaling repressors, and 5 unigenes involved in JA catabolism pathways (Fig. 3a; Table S3). The results revealed that the majority of unigenes encoding JA co-receptors, JA signaling activators, and JA signaling repressors exhibited similar expression changes in the two tulip cultivars (Fig. 3a). Among the unigenes involved in the JA biosynthesis pathway, 52 encoding LOX4 and 34 encoding LOX5 showed expression changes in “Ad Rem” and “Red Power” (Table S3). RNA sequencing results showed that *TgLOX4* (F01.PB33674) and *TgLOX5* (F01.PB63464) had higher FPKM values in “Ad Rem” than in “Red Power”. We then verified the expression level changes in *TgLOX4* and *TgLOX5* by real-time qRT-PCR. The results showed that both genes had significantly higher expression in “Ad Rem” than in “Red Power” at four developmental stages, except for *TgLOX4* at the S4 stage (Fig. 3b–e).

Contrasting expression level changes in *TgLOX4* and *TgLOX5* prompted us to investigate the JA contents of the two tulip cultivars. The results indicated that JA contents increased from S1 to S2 in both cultivars and then decreased from S2 to S4 (Fig. 3f). Although JA content was higher in “Red Power” than in “Ad Rem” at S2, it showed the opposite trend at the other three stages, with 4.9-fold higher JA content in “Ad Rem” than in “Red Power” at S4 (Fig. 3f). There was a continuous increase in MeJA content in “Ad Rem” from S1 to S4, but a decreasing pattern was observed in “Red Power” over the same period (Fig. 3g). These results indicated that the two tulip cultivars exhibited significant changes in JA biosynthesis–related genes and JA accumulation following daughter bulb enlargement.

**Figure 4:**
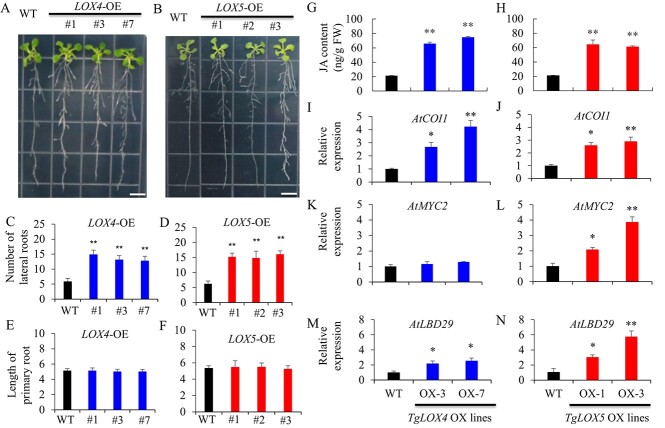
Effect of *TgLOX4* and *TgLOX5* transgenes on root growth and endogenous gene expression in *Arabidopsis* 2 weeks after germination. (a,b) Phenotypic changes in *35::TgLOX4* and *35::TgLOX5* transgenic *Arabidopsis*. (c,d) Number of lateral roots in *35::TgLOX4* and *35::TgLOX5* transgenic and WT *Arabidopsis*. (e,f) Length of primary roots in *35::TgLOX4* and *35::TgLOX5* transgenic and WT *Arabidopsis*. (g,h) JA content in *35::TgLOX4* and *35::TgLOX5* transgenic lines. (i,j) Expression of *AtCOI1.* (k,l) Expression of *AtMYC2*. (m,n) Expression of *AtLBD9*. Values are means ± SEs of three independent experimental replicates (n = 30). Student’s *t*-test was used to analyze statistical significance (^*^P ≤ 0.05, ^**^P ≤ 0.01).

### Ectopic overexpression of *TgLOX4* and *TgLOX5* promoted lateral root growth in *Arabidopsis*

*TgLOX4* and *TgLOX5* were then cloned from “Ad Rem” and “Red Power”. The *TgLOX4* and *TgLOX5* sequences were deposited at NCBI GenBank under accession numbers MW582299, MW582300, MW582301 and MW582302. *TgLOX4* from “Ad Rem” had 2595 nucleotides and encoded 864 amino acids. *TgLOX4* from “Red Power” had 2583 nucleotides and encoded 860 amino acids (Fig. S3). *TgLOX5* from “Ad Rem” had 2574 nucleotides and encoded 857 amino acids. *TgLOX5* from “Red Power” had 2598 nucleotides and encoded 865 amino acids (Fig. S4). Amino acid sequence alignment revealed that both *TgLOX4* and *TgLOX5* showed high similarity between the two tulip cultivars (Fig. S3, S4). Phylogenetic tree analysis showed that *TgLOX4* had high homology with the *LOX4* genes from *Cocos nucifera*, *Elaeis guineensis*, and *Musa balbisiana*, and *TgLOX5* was highly homologous to *LOX5* genes from *Phoenix dactylifera*, *C. nucifera*, and *E. guineensis* (Fig. S5). Tissue-specific expression analysis showed that *TgLOX4* was highly expressed in roots and bulb scales, whereas *TgLOX5* was highly expressed in leaves, roots, and bulb scales (Fig. S6).

*Arabidopsis* lines with ectopic expression of *TgLOX4* and *TgLOX5* were generated. The expression levels of the *TgLOX4* and *TgLOX5* transgenes were detected in the transgenic *Arabidopsis* lines by qRT-PCR (Fig. S7). Our results showed that there were no significant growth differences between transgenic lines overexpressing *TgLOX4* genes from “Ad Rem” or “Red Power” (Fig. S8). Similar phenotypes were obtained from “Ad Rem” and “Red Power” *TgLOX5* overexpression lines (Fig. S8). Therefore, we selected the transgenic lines expressing *TgLOX4* and *TgLOX5* from “Ad Rem” for further analysis.

Interestingly, we observed that *35::TgLOX4* and *35::TgLOX5* transgenic plants displayed significantly more lateral roots compared with the vector wild type (WT) (Fig. 4a–d). However, there were no significant differences in primary root length between the WT and *TgLOX4* or *TgLOX5* transgenic lines (Fig. 4e,f). We then detected JA content in the *TgLOX4* and *TgLOX5* transgenic plants. The results showed that both *TgLOX4* and *TgLOX5* transgenic lines had significantly higher JA content than the WT (Fig. 4g,h). Expression of the JA co-receptor *CORONATINE INSENSITIVE1* (*AtCOI1*) increased 2.6–42-fold in transgenic *Arabidopsis* compared with the WT (Fig. 4i,j). The basic-helix–loop–helix (bHLH) transcription factor (TF) MYC2 functions as a master regulator to activate downstream JA-responsive genes. Overexpression of *TgLOX4* resulted in slightly increased expression of *AtMYC2*, whereas overexpression of the *TgLOX5* transgene significantly enhanced *AtMYC2* expression (Fig. 4k,l). Moreover, expression of *Arabidopsis LATERAL ORGAN BOUNDARIES* (*LOB*) *DOMAIN-CONTAINING PROTEIN* genes (*LBDs*), including *AtLBD13*, *AtLBD14*, *AtLBD16*, *AtLBD18* and *AtLBD29*, was upregulated in *TgLOX4* and *TgLOX5* transgenic *Arabidopsis* (Fig. 4m,n; Fig. S9). These results indicated that ectopic overexpression of *TgLOX4* and *TgLOX5* in *Arabidopsis* activated JA signaling pathways and lateral organ development–related genes.

### Ectopic overexpression of *TgLOX4* and *TgLOX5* promoted leaf growth and branching in *Arabidopsis*

**Figure 5:**
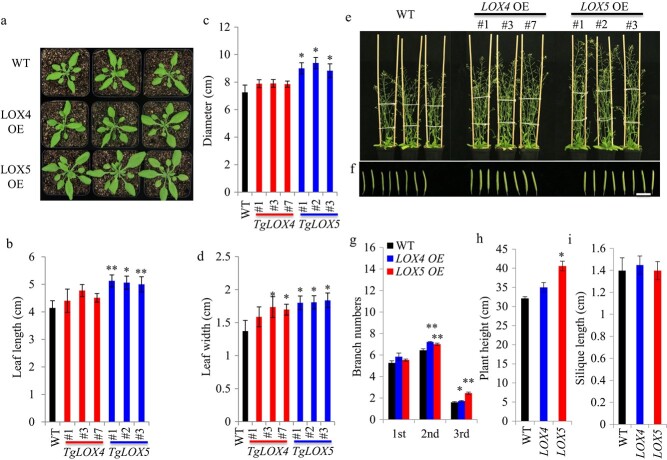
Effect of *TgLOX4* and *TgLOX5* transgenes on aboveground growth of *Arabidopsis*. (a) Vegetative growth of *35::TgLOX4* and *35::TgLOX5* transgenic *Arabidopsis* at 3 weeks after planting. (b–d) Leaf length, rosette diameter, and leaf width of *35::TgLOX4* and *35::TgLOX5* transgenic *Arabidopsis* and the vector WT at 3 weeks after planting. The 7^th^ or 8^th^ leaf with the largest size was measured. (e) Productive growth of *TgLOX4* and *TgLOX5* transgenic *Arabidopsis* at 5 weeks after planting. (f) Siliques of *TgLOX4* and *TgLOX5* transgenic *Arabidopsis* and the vector WT at 5 weeks after planting. Bar = 1 cm. (g–i) Branch numbers, plant height, and silique length of *TgLOX4* and *TgLOX5* transgenic *Arabidopsis* and the vector WT at 5 weeks after planting. The values are means ± SEs of three independent experimental replicates. Student’s *t*-test was used to analyze statistical significance (^*^P ≤ 0.05, ^**^P ≤ 0.01).

After growth in soil for three weeks, there were no significant differences in rosette diameter between *35::TgLOX4* and WT plants, but *35::TgLOX5* transgenic plants showed significantly larger rosette diameters than the WT (Fig. 5a,b). *35::TgLOX5* transgenic plants displayed significantly longer leaf lengths than the WT, whereas the leaf lengths of *35::TgLOX4* transgenic plants were slightly but not significantly longer than those of the WT (Fig. 5c). Two *35::TgLOX4* transgenic lines and all three *35::TgLOX5* transgenic lines exhibited significantly greater leaf widths than the WT (Fig. 5d). In addition, both *35::TgLOX4* and *35::TgLOX5* transgenic plants had significantly more second and third branches (Fig. 5e,g). *35::TgLOX5* transgenic lines also showed significantly higher plant heights than the WT (Fig. 5h). There were no significant differences in silique length between transgenic plants and the WT (Fig. 5f,i). These data showed that *35::TgLOX4* and *35::TgLOX5* transgenes promoted leaf growth and branching in *Arabidopsis*.

### Silencing of *TgLOX4* and *TgLOX5* inhibited tulip daughter bulb growth

**Figure 6:**
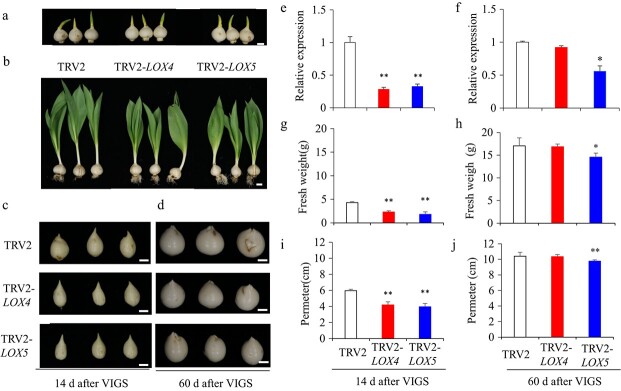
Silencing of *TgLOX4* and *TgLOX5* by VIGS inhibited tulip bulb growth. (a) Mother tulip bulbs used for VIGS infection. (b) Tulip plants after VIGS infection for 14 d. (c) Daughter bulbs after silencing of *TgLOX4* and *TgLOX5* for 14 d. (d) Daughter bulbs after silencing of *TgLOX4* and *TgLOX5* for 60 d. (e,f) Relative expression of two genes in recombinant TRV2-infected tulip bulbs after 14 and 60 d. (g,h) Fresh weights of recombinant TRV2-infected tulip bulbs after 14 and 60 d. (i,j) Perimeter of daughter bulbs in recombinant TRV2-infected tulip bulbs after 14 and 60 d. The tulip cultivar “Ad Rem” was used for virus infection. The values are means ± SEs of three independent experimental replicates (n = 30). Student’s *t*-test was used to analyze statistical significance (^*^P ≤ 0.05, ^**^P ≤ 0.01). Bars = 1 cm.

To further characterize the functions of *TgLOX4* and *TgLOX5* in tulip, we set up a VIGS system using TRV2-*TgLOX4* and TRV2-*TgLOX5* recombinant vectors. Tulip bulbs used for VIGS infection were of uniform size (Fig. 6a). The presence of TRV was verified by genomic PCR (Fig. S10). At 14 days after recombinant vector infection, TRV2-*TgLOX4* and TRV2-*TgLOX5* tulip plants exhibited slower growth compared with the TRV2 controls (Fig. 6b). Daughter bulbs were photographed 14 d and 60 d after VIGS treatments (Fig. 6c,d). Expression analysis of *TgLOX4* and *TgLOX5* in tulip bulbs showed that *TgLOX4* and *TgLOX5* gene expression decreased by 72% and 68% at 14 d and by 8% and 44% at 60 d after infection, respectively (Fig. 6e,f). Fresh weights and perimeters of TRV2-*TgLOX4* and TRV2-*TgLOX5* infected bulbs were significantly lower than those of TRV2 controls 14 d after infection (Fig. 6g,i). TRV2-*TgLOX5* infected bulbs also exhibited significantly lower fresh weights and perimeters than the TRV2 controls at 60 d after infection, but there were no significant differences between TRV2-*TgLOX4* and the TRV2 controls at 60 d (Fig. 6h,j). Therefore, silencing of *TgLOX4* and *TgLOX5* inhibited tulip daughter bulb growth.

### JA promoted tulip bulb growth in *in vitro* cultivation

**Figure 7:**
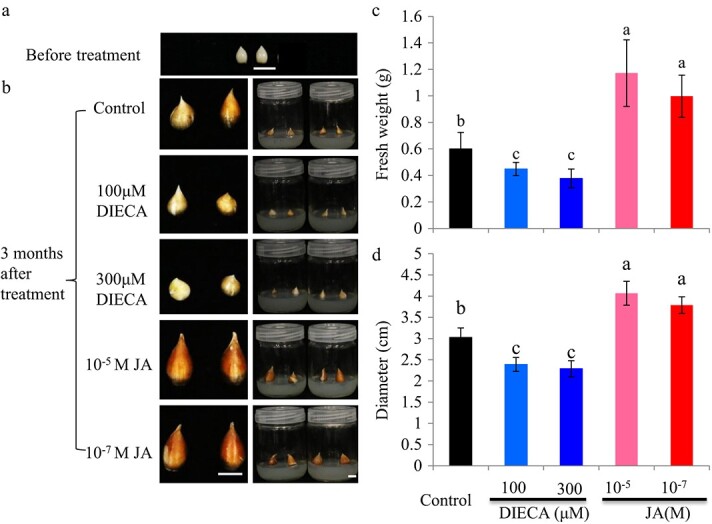
Effects of exogenous JA and the JA biosynthesis inhibitor sodium diethyldithiocarbamate (DIECA) on the growth of tulip daughter bulbs on MS medium. (a) Bulblets with identical sizes separated from mother bulbs after dormancy release. (b) Growth of tulip bulblets on MS medium. The bulblets were sterilized and then cultured on solid MS medium supplemented with JA or DIECA. Photos were taken after 3 months of cultivation. (c) and (d) Fresh weight and diameter of daughter bulbs after JA and DIECA treatments on MS medium. The data were collected after 3 months of cultivation. The values are means ± SEs of three independent experimental replicates (n = 30). Letters indicate statistical significance determined by Duncan’s multiple range test at the *P* ≤ 0.05 level. Bars = 1 cm.

The effects of JA and the JA biosynthesis inhibitor DIECA on growth of tulip daughter bulbs were investigated. Daughter bulbs with identical sizes were separated from mother bulbs after storage at 5°C for 3 months (Fig. 7a). These conditions are used commercially to break tulip dormancy. We observed that JA at 10^−5^ and 10^−7^ M promoted the growth of tulip daughter bulbs, producing significantly higher fresh weights and bulb diameters (Fig. 7b-d). By contrast, DIECA at 100 μM and 300 μM inhibited daughter bulb growth. The fresh weights and bulb diameters of DIECA-treated bulbs were significantly lower than those of control bulbs (Fig. 7b–d). These data indicated that exogenous JA potentially promoted tulip bulb growth under tissue culture conditions.

**Figure 8:**
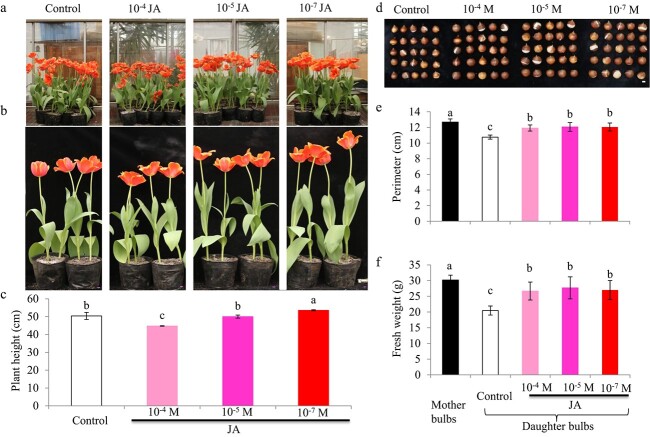
Effects of exogenous JA on the growth of tulips in soil. (a,b) Growth of tulip plants after JA treatments in soil. Photos were taken at 10 days after bloom. Bars = 1 cm. (c) Effect of JA treatment on tulip plant height. (d) Bulbs after treatment with exogenous JA. Bar = 1 cm. Photos were taken at harvest when the aboveground tissues were senescent. (e) Effect of JA treatment on tulip bulb perimeter. (f) Effect of JA treatment on tulip bulb fresh weight. JA solutions were applied as foliar spray at the S2 and S3 stages once every two days for a total of three applications at each stage. The values are means ± SEs of three independent experimental replicates (n = 30). Letters indicate statistical significance determined by Duncan’s multiple range test at the *P* ≤ 0.05 level.

To further investigate the effect of exogenous JA on the growth of tulip, we planted tulip bulbs in a glasshouse and applied JA as a foliar spray at the S2 and S3 stages, three times per stage. Plant height was measured at 10 days after bloom. The results showed that JA at a higher concentration (10^−4^ M) inhibited tulip plant growth, as evidenced by reduced plant height (Fig. 8a–c). JA at 10^−5^ M did not affect plant height, whereas treatment with 10^−7^ M JA significantly promoted tulip plant growth, with significantly greater plant heights compared with the water control (Fig. 8a–c). Interestingly, JA treatments at all three concentrations facilitated the growth of tulip bulbs (Fig. 8d). Perimeters and fresh weights of daughter bulbs were significantly higher than those of control bulbs at the harvest period, but still lower than those of the mother bulbs (Fig. 8e–f). Similar results were also obtained with the cultivar “Red Power”: JA treatments increased bulb perimeter and fresh weight (Fig. S11). These results showed that JA promoted the growth of tulip bulbs in soil.

## Discussion

Tulips, native to the Tien Shan and Pamir-Alay mountains, are one of the most economically important bulbous plants and have been among the top species produced for cut flowers and bedding for many years. The natural propagation rate of tulips is very low [11, 28]. Moreover, the bulb size of tulips is significantly reduced after flowering and generally cannot meet the requirements for flowering in the next season. In the Netherlands and other countries, tulip petals are cut off for tulip bulb production to interrupt reproductive growth, promote the transport of photosynthetic products belowground, and supply the nutrients needed for bulb expansion.

The growth and development of tulip bulbs is a very complex biological process. Bulblet formation is quite similar to axillary bud outgrowth, which is controlled by several hormones in model plants. Hormone signaling is an important factor in the regulation of bulb growth and regeneration [29–31]. JA and MeJA are considered to play important roles in the morphogenesis of storage organs. In *Lycoris radiata*, soluble sugars derived from starch degradation were proposed to be transported from the outer scales to the inner scales, thereby promoting bulblet growth. This process is accompanied by changes in a variety of plant hormones and hormone-responsive genes [29]. The biosynthesis of JA generally occurs in developing and expanding organs. The content of endogenous JA in expanded bulbs is more than three times that in non-expanded bulbs [32]. In this study, we showed that JA content differed significantly among tulip bulbs at four developmental stages (Fig. 3f). It should be pointed out that the JA content was not closely consistent with the changes in *TgLOX4* and *TgLOX5* expression levels (Fig. 3). One possibility is that gene expression changes occur earlier than changes in metabolite contents. Another possibility is that other *TgLOX* genes and JA catabolism–related genes encoding allene oxide synthase are involved in endogenous JA biosynthesis. In addition, “Ad Rem” exhibited higher JA content than “Red Power” at the S3 and S4 stages, which may have contributed to the larger bulb size of “Ad Rem”. JA has been shown to induce and promote bulb formation in onion [33, 34], tulip [27] and *Narcissus* [35] *in vitro* and to significantly increase endogenous methyl jasmonate content during storage organ formation [36]. In tulip, exogenous JA treatment promoted daughter bulb growth under field and tissue-culture conditions (Fig. 7; 8). Accordingly, the JA biosynthesis inhibitor DIECA inhibited tulip bulb enlargement (Fig. 7). JA and MeJA were proposed to be involved mainly in potato tuber development rather than in tuber induction [37, 38]. These data showed that JA is one of the key hormones that control bulb formation and development.

Plant LOXs are involved in diverse functions, including growth and development, stress response, senescence, seed germination, fruit ripening, and synthesis of JA and ABA [39]. *LOX* genes were significantly upregulated by external environmental cues and resulted in the accumulation of JA and MeJA [20, 40]. During the development of potato tubers, JA is metabolized to tuberonic acid (TA) and finally to tuberonic acid glucoside (TAG). TAG is recognized as an endogenous inducer of potato tuber formation [41]. Therefore, LOX derivatives are considered to be key compounds in tuber organogenesis [36]. LOX activity has been found to vary with growth temperature, and the highest LOX activity was found at 15–20°C, when tuber growth was most active [42, 43]. Through transcriptomic profiling analysis, we further observed that the majority of JA biosynthesis–related unigenes, including *TgLOX4* and *TgLOX5*, displayed contrasting changes in the daughter bulbs of two tulip cultivars (Fig. 3). In tomato, *LOX* genes are involved in growth, development and fruit ripening [22]. In tulip, ectopic overexpression of *TgLOX4* and *TgLOX5* improved the growth of underground roots and aboveground stems and leaves (Figs. 4, 5). Silencing of *TgLOX4* and *TgLOX5* in tulip repressed growth of tulip plants and bulbs (Fig. 6), indicating that these two LOX genes are involved in tulip bulb enlargement. These results are consistent with data from potato in which reduced transcript levels of potato *LOX1* inhibited LOX activity, resulting in reduced tuber yield, decreased average tuber size, and disruption of tuber formation [19]. Persimmon (*Diospyros kaki*) *LOX3* transgenic *Arabidopsis* exhibited faster root growth under osmotic stress conditions compared with the WT [44], whereas mutation of maize *lox3* reduced root length and plant height [45]. In this study, *Arabidopsis TgLOX4* and *TgLOX5* overexpression lines exhibited more lateral roots and branches and greater plant height compared with the WT, indicating that *TgLOX* genes are involved in stem and root development, at least in *Arabidopsis*. In addition, VIGS data showed that silencing of *TgLOX4* and *TgLOX5* repressed tulip bulb growth. Therefore, data from *Arabidopsis* and tulip were consistent, partially illuminating the roles played by *TgLOX4* and *TgLOX5* in tulip bulb growth. However, we cannot rule out the possibility that several other LOXs function as JA biosynthetic enzymes. The functions of TgLOX4 and TgLOX5 in the JA biosynthesis pathway are worthy of further discussion.

Taken together, transcriptomic analyses showed that hormone-related pathways were extensively changed during tulip bulb growth and development. Ectopic overexpression of the tulip lipoxygenase genes *TgLOX4* and *TgLOX5* in *Arabidopsis* increased endogenous JA content and improved plant and root growth, whereas silencing of these genes inhibited tulip bulb development. We propose that *TgLOX4* and *TgLOX5* enhance JA biosynthesis, activate JA signaling pathways, and possibly promote tulip bulb growth and development by upregulating the expression of *LBD* and JA-responsive genes (Fig. S10). In addition, the effect of MeJA on bulb development may be combined with those of other hormones [26]. External application of MeJA can reduce the content of hormones that are not conducive to bulb expansion, such as GA1, GA3 and ABA, and increase the content of IAA, which is conducive to bulb expansion, thus better promoting the formation and expansion of renewed bulbs [46]. Moreover, the observation that LOX regulates tuber formation by directly interacting with light and growing temperature [42, 43] suggests that it may be an important downstream signaling molecule in photoperiod-controlled signaling pathway(s). The regulatory networks among different hormones during the development of storage organs are worthy of further investigation.

## Materials and methods

### Plant materials and growth conditions

Sixty-six tulip cultivars were used in this study. All mother bulbs were imported from the Netherlands and planted in Wuhan, China for investigation of bulb perimeter size. In total, 50 daughter bulbs were investigated and three replicates were used. Two tulip cultivars with contrasting bulb sizes, “Ad Rem” and “Red Power”, were used for further study. The mother bulbs were imported from the Netherlands by Shangu Horticultural Company (Beijing) and stored at 5°C for 12 weeks. The tulip bulbs were planted in a glasshouse at the Ornamental Plants Research Farm of Huazhong Agricultural University (Wuhan, China). The conditions in the greenhouse were maintained at 20°C day/15°C night, with a relative humidity of 60–70% and a 16-h light/8-h dark photoperiod.

Moreno-Pachon et al. classified the developmental stages of tulip plants and bulblets from the storage period (October to December) through the growing season (February to July) under field conditions in the Netherlands [10]. In this study, a cold forcing treatment was performed in a cold room maintained at 5°C for 12 weeks. All cold-treated tulip bulbs were planted in February, bloomed in March, and senesced in April; the bulbs were harvested in May under greenhouse conditions in Wuhan, China. Bulblet samples from the following four developmental stages were collected for further analysis: S1, bulblets inside the mother bulbs after dormancy release in early February; S2, bulblets from tulip plants with green buds 4–5 cm in length in early March; S3, bulbs one week after full bloom in middle (“Red Power”) or later (“Ad Rem”) March; and S4, bulbs from plants that were senescent in April. These four stages are nearly equivalent to the stages of Dec, Mar, Apr, and Jun described by Moreno-Pachon et al. [10].

The *Arabidopsis thaliana* Columbia-0 (Col-0) ecotype was used in this study to generate transgenic plants. *Arabidopsis* seeds were sterilized for 4 min using 2% (v/v) sodium hypochlorite (NaClO) containing 0.1% (v/v) Triton X-100. The seeds were then washed five times with sterile water. Seeds were stored at 4°C for 5 days under dark conditions for vernalization. After planting on MS plates, all seeds were cultured in a growth chamber maintained at 22 ± 1°C with 60% relative humidity and a 16-h light/8-h dark photoperiod. The chamber was supplemented with 100 μmol photons m^−2^ s^−1^ light intensity.

### Determination of JA content

Plant tissues were frozen with liquid nitrogen and ground into fine powder in a mortar. Approximately 100-mg samples were transferred to 2-mL tubes containing 1 mL extraction solvent (2-propanol: H_2_O: concentrated HCl = 2:1:0.002, v/v/v). Then 100 μL of the 2H-JA working solution was added as an internal standard. The samples were mixed well and centrifuged at 4°C and 13 000 *g* for 5 min. After centrifugation, 900 μL of the solvent from the lower phase was transferred and concentrated using a nitrogen evaporator with nitrogen flow. The samples were re-dissolved in 0.1 mL methanol, and 50 μL of sample solution was injected into a reverse-phase C18 Gemini HPLC column for HPLC-ESI-MS/MS analysis.

### RNA sequencing analysis of tulip bulbs at four developmental stages

Tulip bulbs of two cultivars at four developmental stages were harvested for RNA isolation using a plant RNA purification kit (Tiangen, Beijing, China). A NanoDrop 2000 spectrophotometer (Thermo, USA) and a Bioanalyzer 2100 system (Agilent Technologies, USA) were used to assess RNA purity and integrity, respectively. A total amount of 1 μg RNA per sample was used for cDNA library construction with the NEBNext Ultra RNA Library Prep Kit for Illumina (NEB, USA) following the manufacturer’s recommendations. The libraries were sequenced on an Illumina HiSeq platform, and 150-bp paired-end reads were generated. Clean data (clean reads) were obtained by removing low-quality reads and reads that contained adapters and poly-N from the raw data. Trinity was used for transcriptome assembly based on the left.fq and right.fq files [47]. Gene functions of the tulip unigenes were annotated using the following databases: NR (NCBI non-redundant protein sequences), Swiss-Prot (a manually annotated and reviewed protein sequence database), KOG/COG/eggNOG (Clusters of Orthologous Groups of proteins), Pfam (Protein family), KEGG (Kyoto Encyclopedia of Genes and Genomes) and GO (Gene Ontology). HTSeq v0.6.1 was used to count the read numbers mapped to each gene. The FPKM value (Fragments Per Kilobase of transcript sequence per Million base pairs sequenced) for each unigene was calculated based on its length and mapped read count. Differential gene expression between combinations of cultivars and developmental stages was analyzed using the DESeq R package (1.18.0). The raw data have been deposited to the NCBI Gene Expression Omnibus (GEO) with the accession number GSE167530.

### Clustering analysis

Hierarchical clustering analysis was performed using the CLUSTER 3.0 program (http://bonsai.hgc.jp/~mdehoon/software/cluster/) with an uncentered matrix and complete linkage methods [48]. The resulting tree figures were displayed using the software package Java Treeview (http://jtreeview.sourceforge.net/) as described previously [49].

### MapMan pathway enrichment analysis

Differentially expressed tulip unigenes were annotated based on their *Arabidopsis* homologs. Corresponding Arabidopsis Genome Initiative (AGI) locus codes for differentially expressed unigenes were used as input to the Classification SuperViewer Tool (http://bar.utoronto.ca/ntools/cgi-bin/ntools_classification_superviewer.cgi) for pathway enrichment analyses [50]. MapMan (http://mapman.gabipd.org/home) was selected as a classification source. The normalized frequency (NF) was calculated as described previously [49]: NF = sample frequency of each category in each sample/background frequency of each category.

### Gene cloning, plasmid construction and gene transformation

Based on tulip RNA sequencing data (GEO database accession number GSE167530), the full-length sequences of F01.PB33674 (*TgLOX4*) and F01.PB63464 (*TgLOX5*) were identified. The coding regions of *TgLOX4* and *TgLOX5* were amplified using the specific primers listed in Table S4. The open reading frames (ORFs) of *TgLOX4* and *TgLOX5* were cloned into the pCAMBIA1300 vector using the XbaI and KpnI restriction sites, and the resulting plasmids were introduced into *Agrobacterium tumefaciens* strain GV3101. Transgenic *Arabidopsis* lines were generated by the floral-dip method [51]. *TgLOX4* and *TgLOX5* transgenic plants were screened and verified by qPCR analysis with the primers listed in Table S4.

### Phylogenetic analysis and sequence alignment

Amino acids sequence alignment of *TgLOX4* and *TgLOX5* and their closest orthologs was performed using BioEdit (Tom Hall, North Carolina State University, USA). Multiple protein sequence alignments and phylogenetic tree construction for *TgLOX4* and *TgLOX5* were performed using MEGA 7 with the maximum likelihood method.

### Measurement of root length and lateral root number

Transgenic (T2 generation) and wild-type *Arabidopsis* were planted directly in soil for leaf and stem measurements. Each pot contained only one seedling at 1 week after planting. Leaf length, leaf width and seedling diameter were measured at 21 days after planting, and branch number, plant height and silique length were measured at 42 days after planting.

To investigate root length and lateral root number, wild-type and transgenic *Arabidopsis* seeds were sown on MS plates. One-week-old seedlings of identical size were transferred to fresh MS plates. Primary root length was measured and lateral root numbers were counted after 7 d of growth. For each genotype, three replicates of at least 30 seedlings each were measured, and the whole experiment was repeated three times.

### Silencing of target genes in tulip

The expression of *TgLOX4* and *TgLOX5* genes was silenced through virus-induced gene silencing (VIGS) as described by Zhong et al. [52] and Wang et al. [53]. A 394-bp fragment of *TgLOX4* and a 394-bp fragment of *TgLOX5* were amplified by PCR and inserted into the pTRV2 vector to generate the pTRV2-*TgLOX4* and pTRV2-*TgLOX5* constructs, respectively. *A. tumefaciens* strain GV3101 was used for construct transformation. Tulip bulbs were immersed in infiltration buffer containing *Agrobacterium* cells transformed with equal amounts of pTRV1 and pTRV2 or pTRV2-target genes. Tulip bulbs submerged in the bacterial suspension were infiltrated under a vacuum at 0.8 MPa for 30 min to promote infection efficiency. After infiltration, the bulbs were kept in the dark at 22°C for 48 h, then planted in a greenhouse at 22°C with a relative humidity of 60–70% and a 16-h light/8-h dark cycle.

### Real-time qRT-PCR analysis

Tulip bulbs at four developmental stages and whole seedlings of 2-week-old transgenic *TgLOX4*, *TgLOX5*, and WT *Arabidopsis* were collected for qPCR analysis. Total RNA was extracted from tulip bulbs and *Arabidopsis* using the EASYspin Plus Complex Plant RNA Kit (Vazyme, Nanjing China). Equal amounts (1 μg) of total RNA were used for reverse transcription with the HiScript II 1st Strand cDNA Synthesis Kit (Vazyme, Nanjing, China) following the manufacturer’s instructions. *AtACT2* (AT3G18780) was used as the reference gene for *Arabidopsis*, and tulip *TgACTIN* (unigene ID PB13161) was used as the reference gene for tulip. The web tool GenScript (https://www.genscript.com/ssl-bin/app/primer) was used to design real-time qRT-PCR primers. The relative transcription levels were calculated using the 2^−ΔΔCt^ method [54]. Primer sequences are listed in Table S4.

### Effects of JA and the JA biosynthesis inhibitor DIECA on growth of bulblets *in vitro*

“Ad Rem” daughter bulbs (axillary buds) were separated from mother bulbs after three months of storage at 5°C. The bulblets were sterilized in 70% ethanol for 1 min, then soaked in 20% (v/v) sodium hypochlorite (NaClO) for 20 min and finally washed with sterilized water 5 times. The sterile buds were cultured on solid MS medium containing 60 g l^−1^ sucrose, 1.0 g l^−1^ casein hydrolysate, 1.0 mg l^−1^ thiamine and 200 mg l^−1^ L-Gln. Different concentrations of JA and the JA biosynthesis inhibitor DIECA were added to the MS medium. After incubation at 22°C with a 16-h light/8-h dark cycle for three months, fresh weights and perimeters of daughter bulbs were measured.

For exogenous JA treatment, JAs at the indicated concentrations and water (control) were sprayed on both sides of the plant leaves. Three concentrations (10^−4^ M, 10^−5^ M, and 10^−7^ M) of JA solution were used based on preliminary results. Both the water control and the JA solutions were applied as foliar spray at the S2 and S3 stages once every two days, three times in total at each stage. For each replicate (30 plants), 2 L of solution containing the indicated concentration of JA or water was used.

## Statistical analysis

All experiments in this study were performed three times, and the results shown are mean ± SEs (n = 3) of each replicate. At least 50 bulbs or plants were used for each treatment. Duncan’s multiple range test (DMRT) was used to assess differences between the means. Different letters above the columns in each figure indicate significant differences at *P < 0.05.

## Acknowledgments

This research was supported by the National Key Research and Development Program (2020YFD1000402) and the Huazhong Agricultural University Scientific & Technological Self-innovation Foundation to Zhulong Chan (2662016QD026 and 2662016RC010) and Yanping Wang (2662020YLPY010).

## Author contributions

Q.S. and Z.C. conceived the experiments. Q.S. and B.Z. conducted the experiments. C.Y. and W.W. helped with data collection. Q.S. and Z.C. wrote the manuscript. Y.W. and L.X. guided the research and revised the manuscript. All authors read and approved the manuscript.

## Data availability

The raw RNA sequencing data have been deposited at the NCBI Gene Expression Omnibus (GEO) under accession number GSE167530. The sequences of *TgLOX4* and *TgLOX5* from two tulip cultivars have been deposited at NCBI GenBank under accession numbers MW582299, MW582300, MW582301 and MW582302.

## Conflict of interest statement

The authors declare no competing interests.

## Supplementary data

Supplementary data is available at *Horticulture Research* online.

## Supplementary Material

Web_Material_uhac006
