# Supplementary material for: Jasmonic acid biosynthetic genes TgLOX4 and TgLOX5 are involved in daughter bulb development in tulip (Tulipa gesneriana)
Source: Hortic Res. 2022 Feb 11;9:uhac006. doi: 10.1093/hr/uhac006 (PMC8947238; doi:10.1093/hr/uhac006)
Supplement: Web_Material_uhac006 [file web_material_uhac006.zip › Supplementary Figs. 1-11.pptx]

## Slide 1
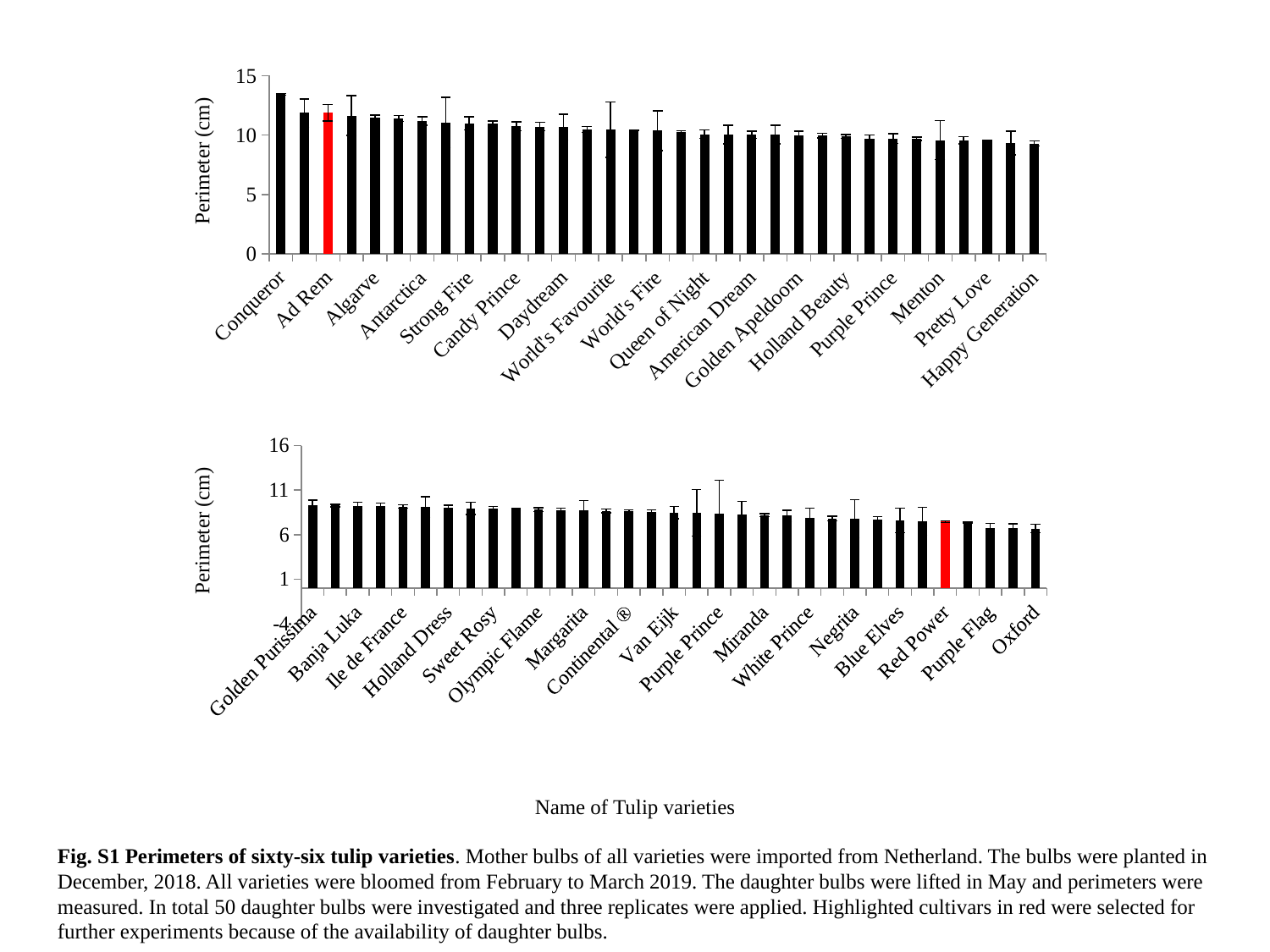

### Chart
| Category | |
|---|---|
| Conqueror | 13.426666666666668 |
| Temple of Beauty | 11.8655555555556 |
| Ad Rem | 11.8933333333333 |
| Banja Luka | 11.643333333333333 |
| Algarve | 11.453333333333333 |
| Spryng | 11.396666666666667 |
| Antarctica | 11.183333333333332 |
| Red Impression | 11.065555555555555 |
| Strong Fire | 10.984444444444444 |
| Pretty Woman | 10.936666666666667 |
| Candy Prince | 10.75111111111111 |
| Pink Sky | 10.71888888888889 |
| Daydream | 10.683333333333332 |
| Parade | 10.483333333333334 |
| World's Favourite | 10.448888888888888 |
| King's Orange | 10.39 |
| World's Fire | 10.37 |
| Orange Queen | 10.270000000000001 |
| Queen of Night | 10.074444444444445 |
| Darwisnow | 10.041111111111112 |
| American Dream | 10.040000000000001 |
| Friendship | 10.032222222222222 |
| Golden Apeldoorn | 9.99 |
| Verandi | 9.956666666666669 |
| Holland Beauty | 9.887777777777778 |
| Blue Diamond | 9.723333333333334 |
| Purple Prince | 9.705555555555557 |
| Miss Rose | 9.693333333333335 |
| Menton | 9.575555555555555 |
| Mystic van Eijk | 9.564444444444446 |
| Pretty Love | 9.459999999999999 |
| Royal Ten | 9.326666666666666 |
| Happy Generation | 9.293333333333335 |Perimeter (cm)
### Chart
| Category | |
|---|---|
| Golden Purissima | 9.272222222222222 |
| Madame Lefeber | 9.26 |
| Banja Luka | 9.241111111111111 |
| Yellow Springgreen | 9.223333333333334 |
| Ile de France | 9.143333333333333 |
| Jumbo Pink | 9.118888888888888 |
| Holland Dress | 9.01 |
| Orange Brilliant | 8.950000000000001 |
| Sweet Rosy | 8.94 |
| Salmon Impression | 8.834444444444445 |
| Olympic Flame | 8.793333333333335 |
| Jaap Groot | 8.73666666666667 |
| Margarita | 8.723333333333334 |
| Christmas Pearl | 8.656666666666666 |
| Continental ® | 8.596666666666666 |
| Durian | 8.583333333333334 |
| Van Eijk | 8.47 |
| Sapporo | 8.444444444444445 |
| Purple Prince | 8.313333333333334 |
| Purissima | 8.243333333333334 |
| Miranda | 8.186666666666667 |
| Apeldoorn | 8.125555555555557 |
| White Prince | 7.900000000000001 |
| Judith Leyster | 7.796666666666667 |
| Negrita | 7.780000000000001 |
| Purple Doll | 7.663333333333334 |
| Blue Elves | 7.598888888888889 |
| Flaming Youth | 7.491111111111111 |
| Red Power | 7.4666666666667 |
| Strong Gold | 7.336666666666666 |
| Purple Flag | 6.752222222222223 |
| Purple Dream | 6.715555555555556 |
| Oxford | 6.69 |Perimeter (cm)
Name of Tulip varieties
Fig. S1 Perimeters of sixty-six tulip varieties. Mother bulbs of all varieties were imported from Netherland. The bulbs were planted in December, 2018. All varieties were bloomed from February to March 2019. The daughter bulbs were lifted in May and perimeters were measured. In total 50 daughter bulbs were investigated and three replicates were applied. Highlighted cultivars in red were selected for further experiments because of the availability of daughter bulbs.

## Slide 2
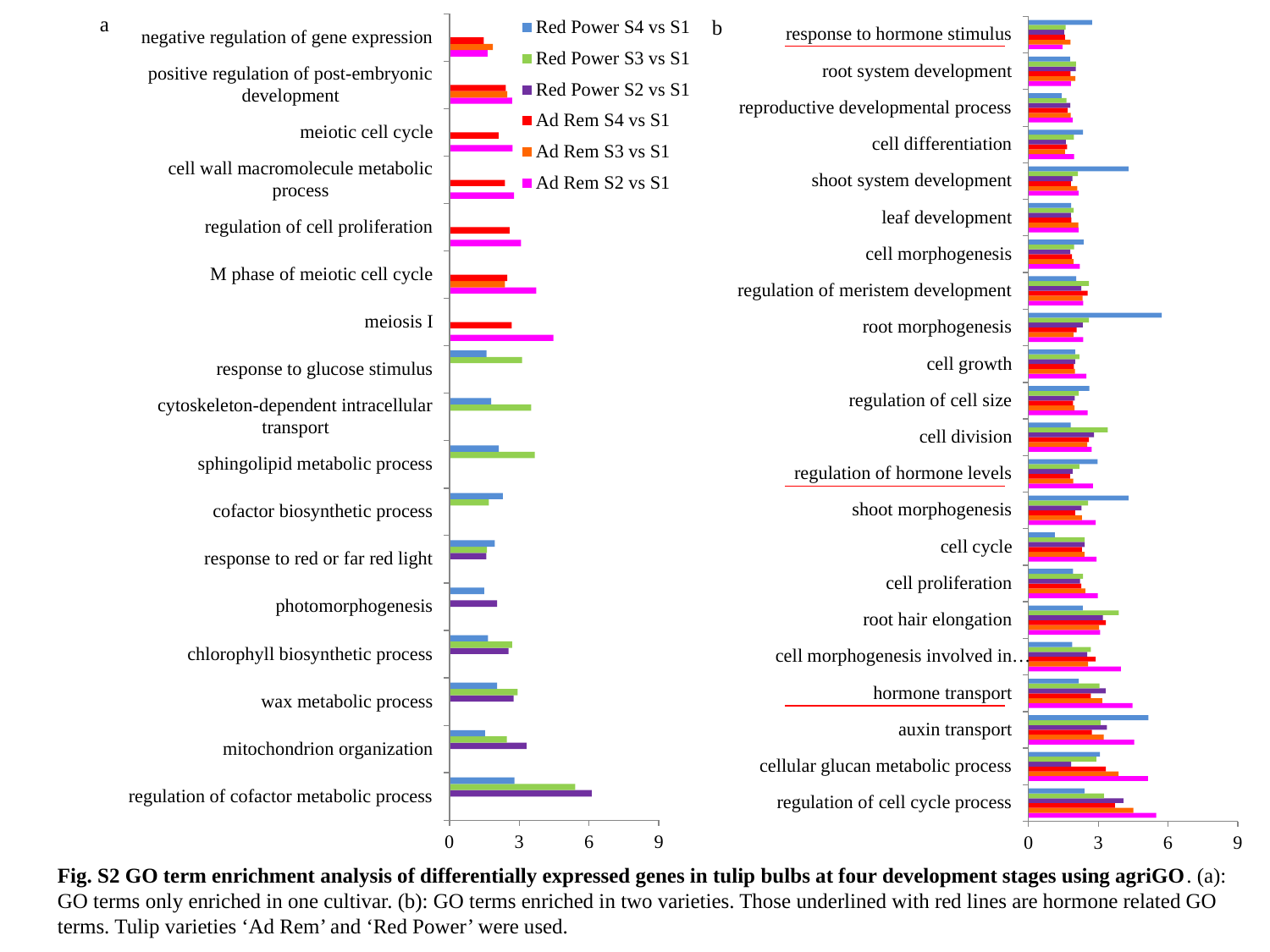

a
b
Fig. S2 GO term enrichment analysis of differentially expressed genes in tulip bulbs at four development stages using agriGO. (a): GO terms only enriched in one cultivar. (b): GO terms enriched in two varieties. Those underlined with red lines are hormone related GO terms. Tulip varieties ‘Ad Rem’ and ‘Red Power’ were used.

## Slide 3
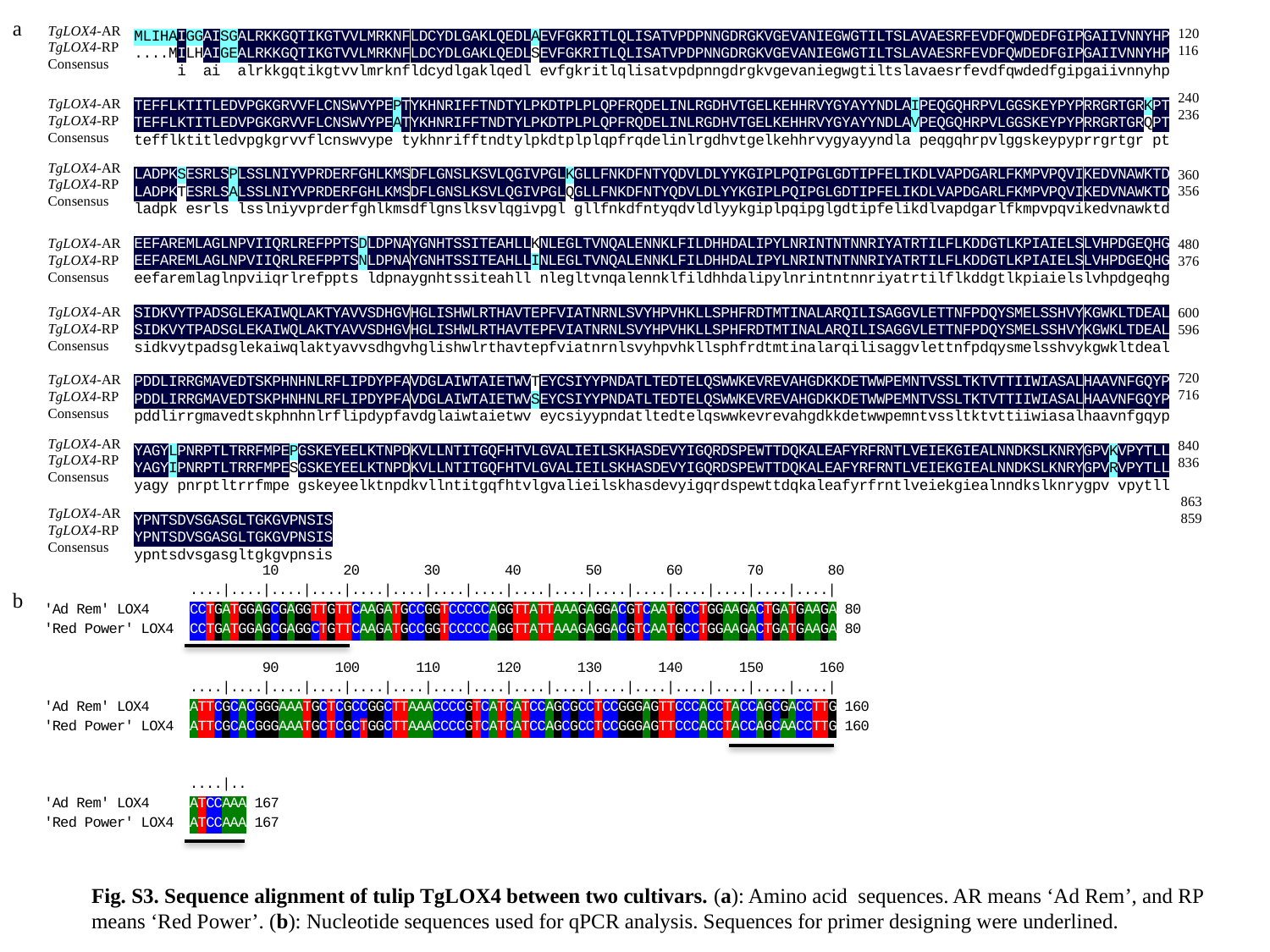

a
TgLOX4-AR
TgLOX4-RP
Consensus
120
116
240
236
TgLOX4-AR
TgLOX4-RP
Consensus
TgLOX4-AR
TgLOX4-RP
Consensus
360
356
TgLOX4-AR
TgLOX4-RP
Consensus
480
376
TgLOX4-AR
TgLOX4-RP
Consensus
600
596
720
716
TgLOX4-AR
TgLOX4-RP
Consensus
TgLOX4-AR
TgLOX4-RP
Consensus
840
836
863
859
TgLOX4-AR
TgLOX4-RP
Consensus
b
Fig. S3. Sequence alignment of tulip TgLOX4 between two cultivars. (a): Amino acid sequences. AR means ‘Ad Rem’, and RP means ‘Red Power’. (b): Nucleotide sequences used for qPCR analysis. Sequences for primer designing were underlined.

## Slide 4
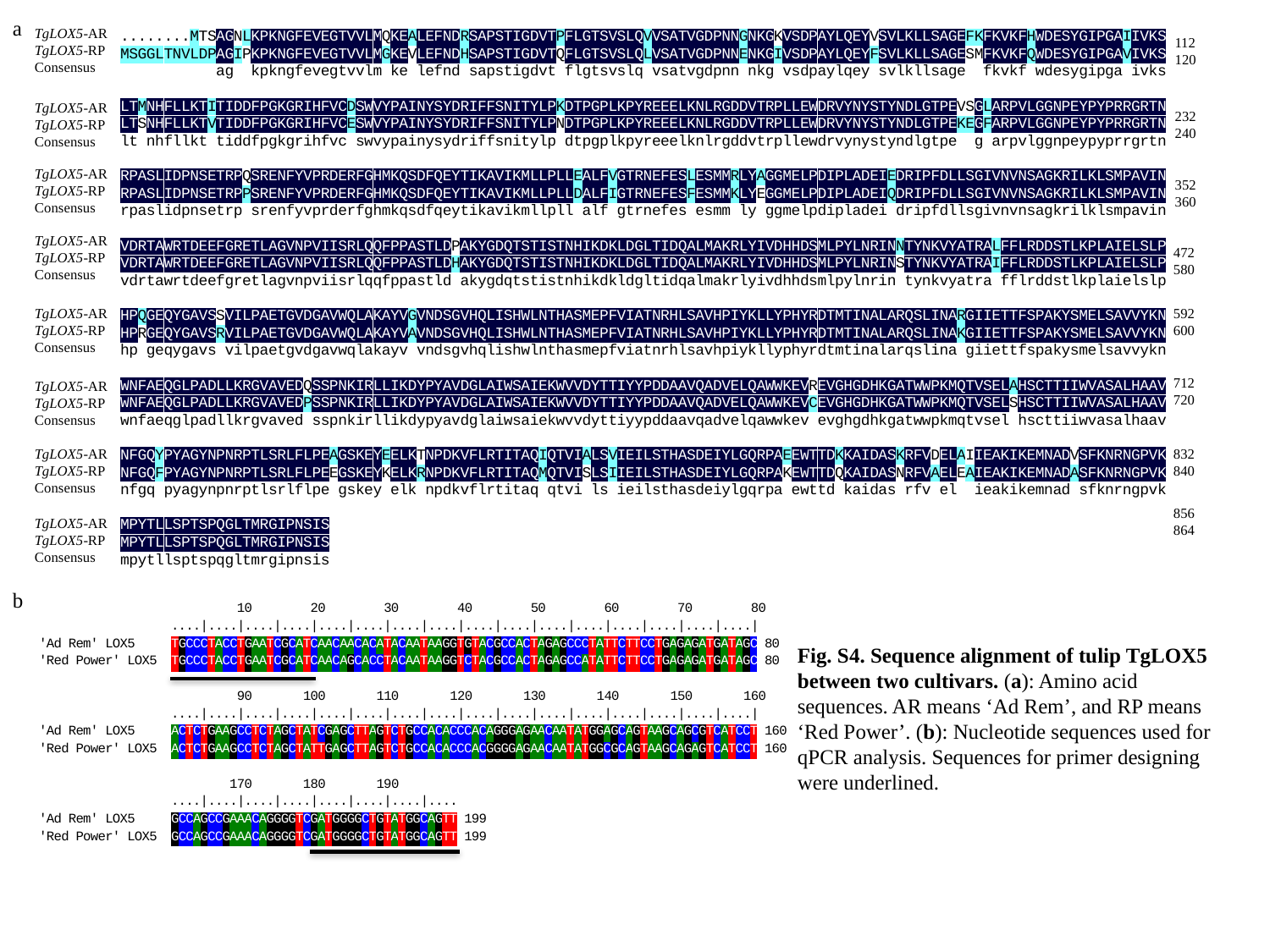

a
TgLOX5-AR
TgLOX5-RP
Consensus
112
120
TgLOX5-AR
TgLOX5-RP
Consensus
232
240
TgLOX5-AR
TgLOX5-RP
Consensus
352
360
TgLOX5-AR
TgLOX5-RP
Consensus
472
580
592
600
TgLOX5-AR
TgLOX5-RP
Consensus
712
720
TgLOX5-AR
TgLOX5-RP
Consensus
832
840
TgLOX5-AR
TgLOX5-RP
Consensus
856
864
TgLOX5-AR
TgLOX5-RP
Consensus
b
Fig. S4. Sequence alignment of tulip TgLOX5 between two cultivars. (a): Amino acid sequences. AR means ‘Ad Rem’, and RP means ‘Red Power’. (b): Nucleotide sequences used for qPCR analysis. Sequences for primer designing were underlined.

## Slide 5
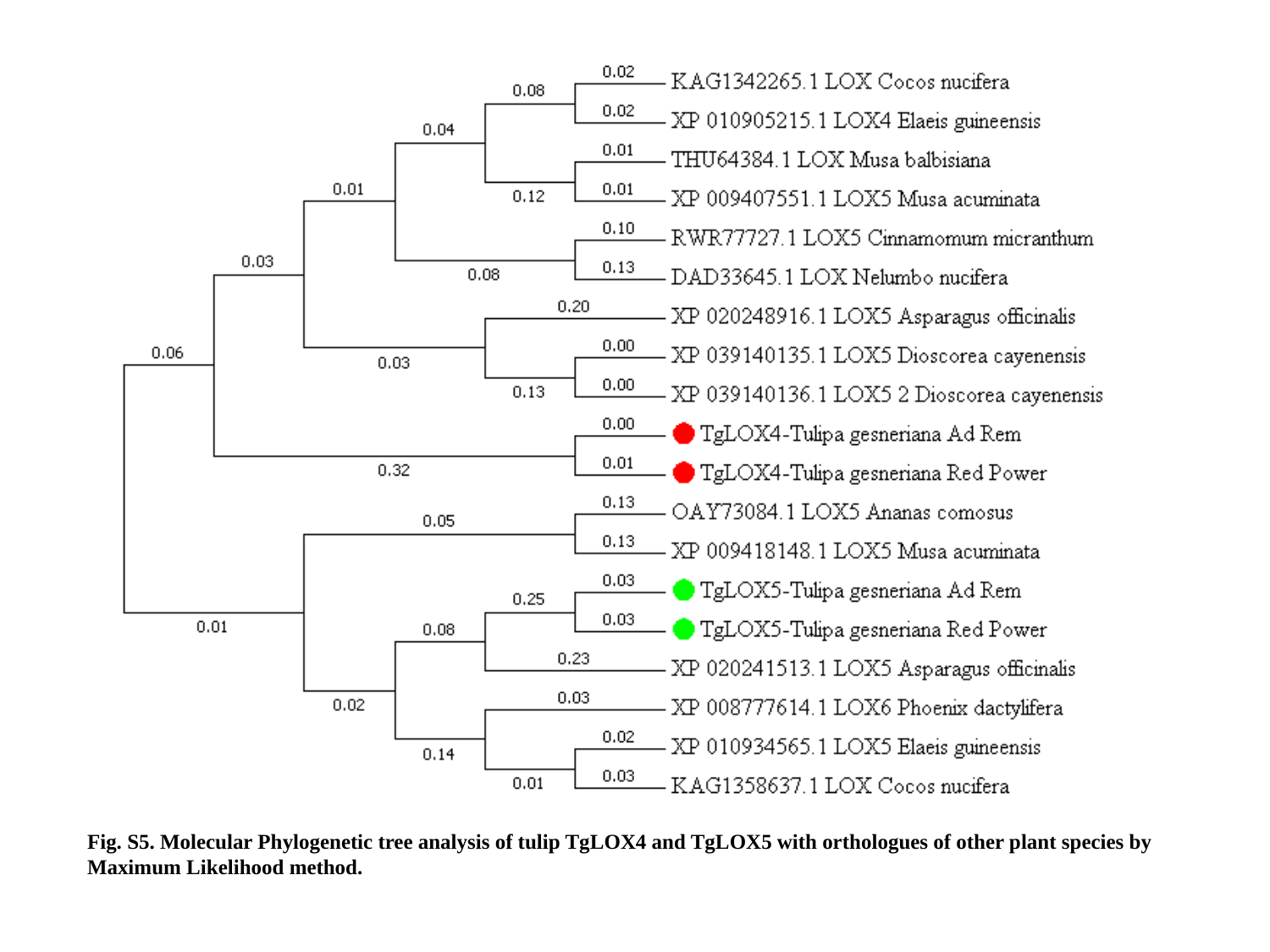

Fig. S5. Molecular Phylogenetic tree analysis of tulip TgLOX4 and TgLOX5 with orthologues of other plant species by Maximum Likelihood method.

## Slide 6
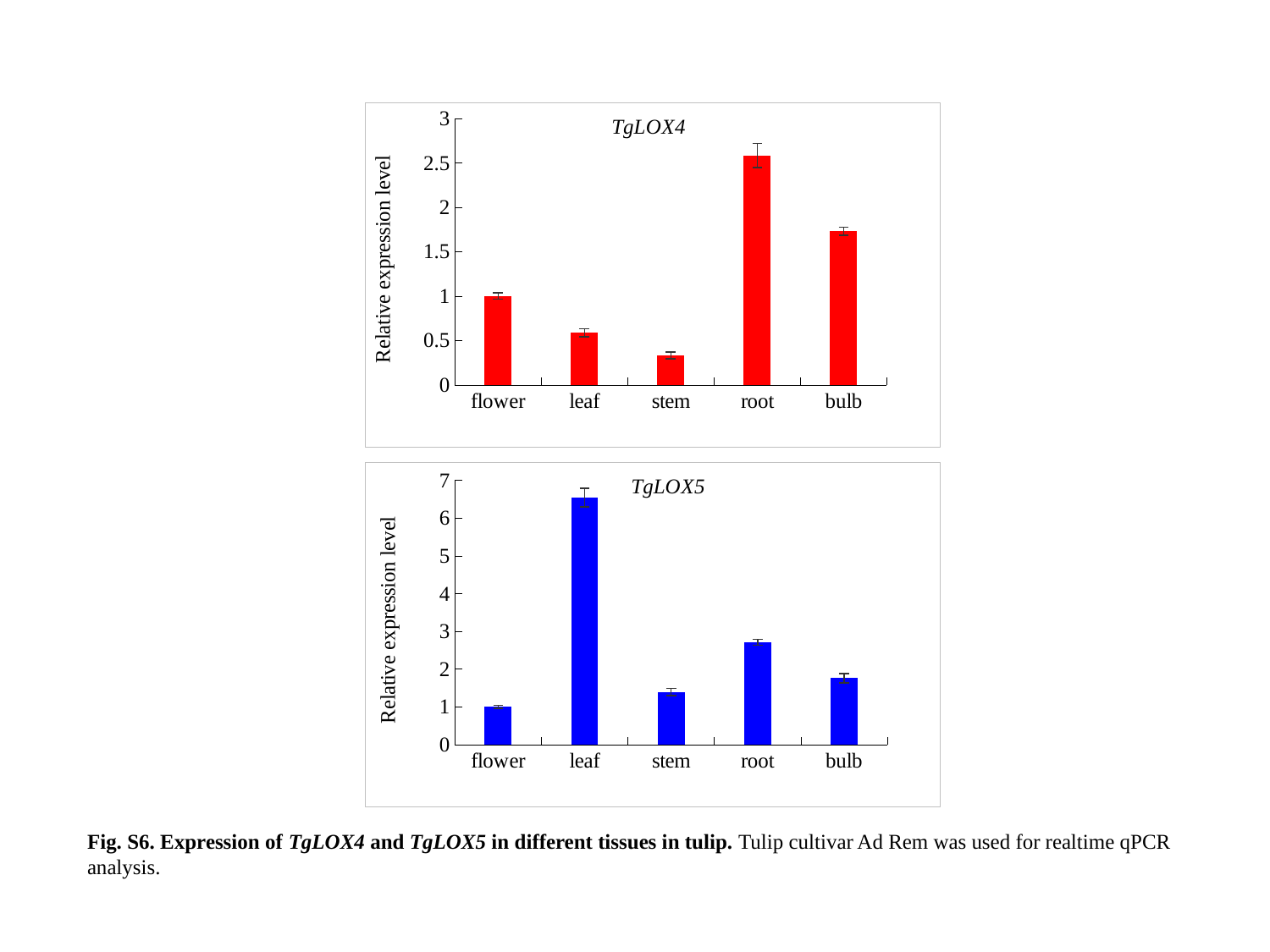

### Chart: TgLOX4
| Category | LOX4 |
|---|---|
| flower | 1.0006091834248054 |
| leaf | 0.58617892887508 |
| stem | 0.33284749906249417 |
| root | 2.581413051737482 |
| bulb | 1.7299513642969842 |
### Chart: TgLOX5
| Category | LOX5 |
|---|---|
| flower | 1.0006948333322507 |
| leaf | 6.545711769560708 |
| stem | 1.3972117450181392 |
| root | 2.708821391423031 |
| bulb | 1.7616803674340016 |Fig. S6. Expression of TgLOX4 and TgLOX5 in different tissues in tulip. Tulip cultivar Ad Rem was used for realtime qPCR analysis.

## Slide 7
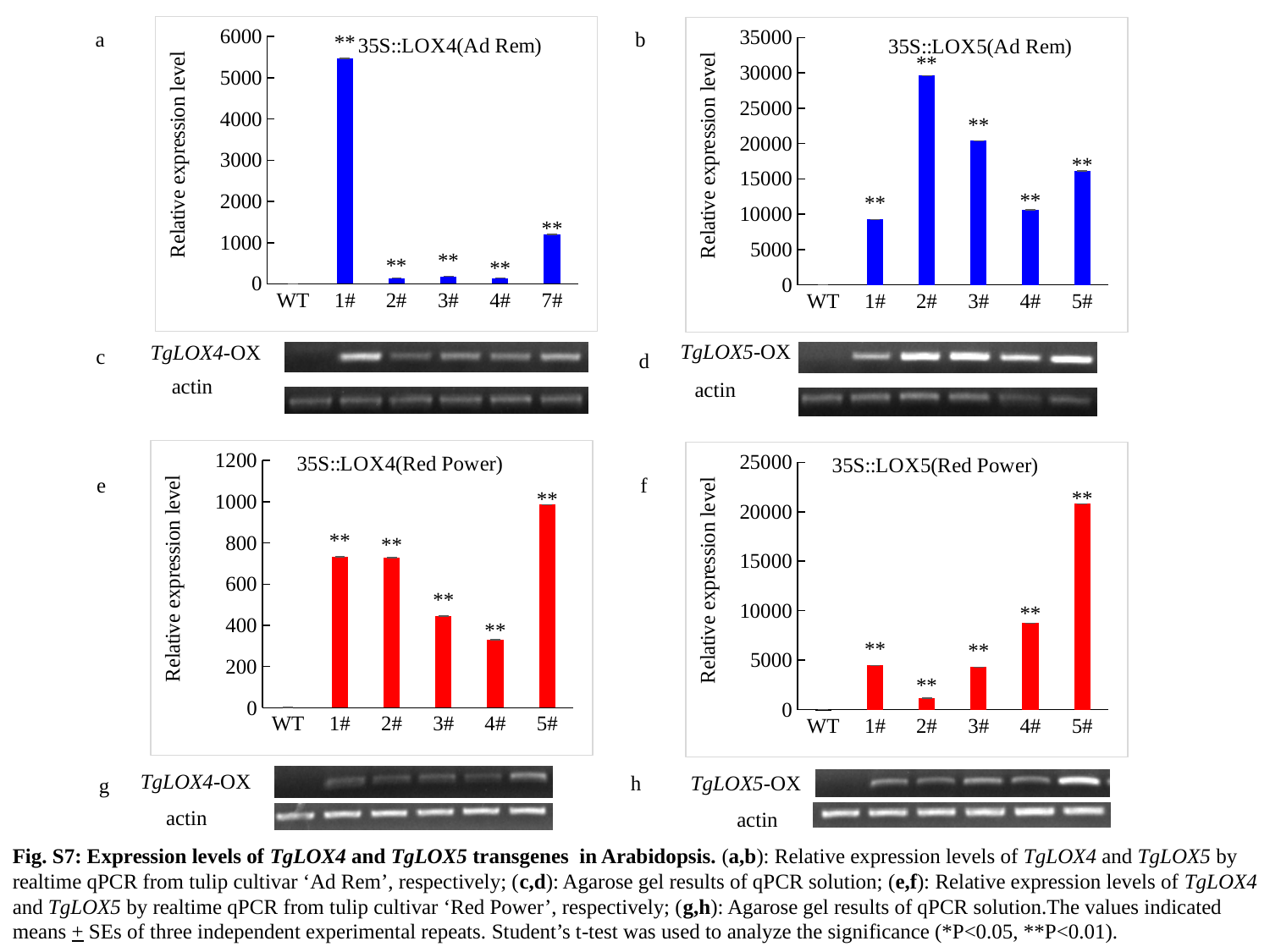

### Chart: 35S::LOX4(Ad Rem)
| Category | |
|---|---|
| WT | 1.0 |
| 1# | 5468.95835749824 |
| 2# | 140.568074542014 |
| 3# | 180.636946760266 |
| 4# | 140.143882657516 |
| 7# | 1200.69925848973 |
### Chart: 35S::LOX5(Ad Rem)
| Category | |
|---|---|
| WT | 1.0 |
| 1# | 9229.02024852979 |
| 2# | 29589.9264297792 |
| 3# | 20362.6100083692 |
| 4# | 10579.4539596223 |
| 5# | 16127.5173420652 |a
b
TgLOX5-OX
TgLOX4-OX
c
d
actin
actin
### Chart: 35S::LOX4(Red Power)
| Category | |
|---|---|
| WT | 1.0 |
| 1# | 732.846079622181 |
| 2# | 729.132612928624 |
| 3# | 444.7171855184 |
| 4# | 329.647086127705 |
| 5# | 985.105042724785 |
### Chart: 35S::LOX5(Red Power)
| Category | |
|---|---|
| WT | 1.0 |
| 1# | 4466.1085130595 |
| 2# | 1161.26871325999 |
| 3# | 4293.90969142081 |
| 4# | 8732.35158751262 |
| 5# | 20799.017947768 |f
e
TgLOX4-OX
h
TgLOX5-OX
g
actin
actin
Fig. S7: Expression levels of TgLOX4 and TgLOX5 transgenes in Arabidopsis. (a,b): Relative expression levels of TgLOX4 and TgLOX5 by realtime qPCR from tulip cultivar ‘Ad Rem’, respectively; (c,d): Agarose gel results of qPCR solution; (e,f): Relative expression levels of TgLOX4 and TgLOX5 by realtime qPCR from tulip cultivar ‘Red Power’, respectively; (g,h): Agarose gel results of qPCR solution.The values indicated means + SEs of three independent experimental repeats. Student’s t-test was used to analyze the significance (*P<0.05, **P<0.01).

## Slide 8
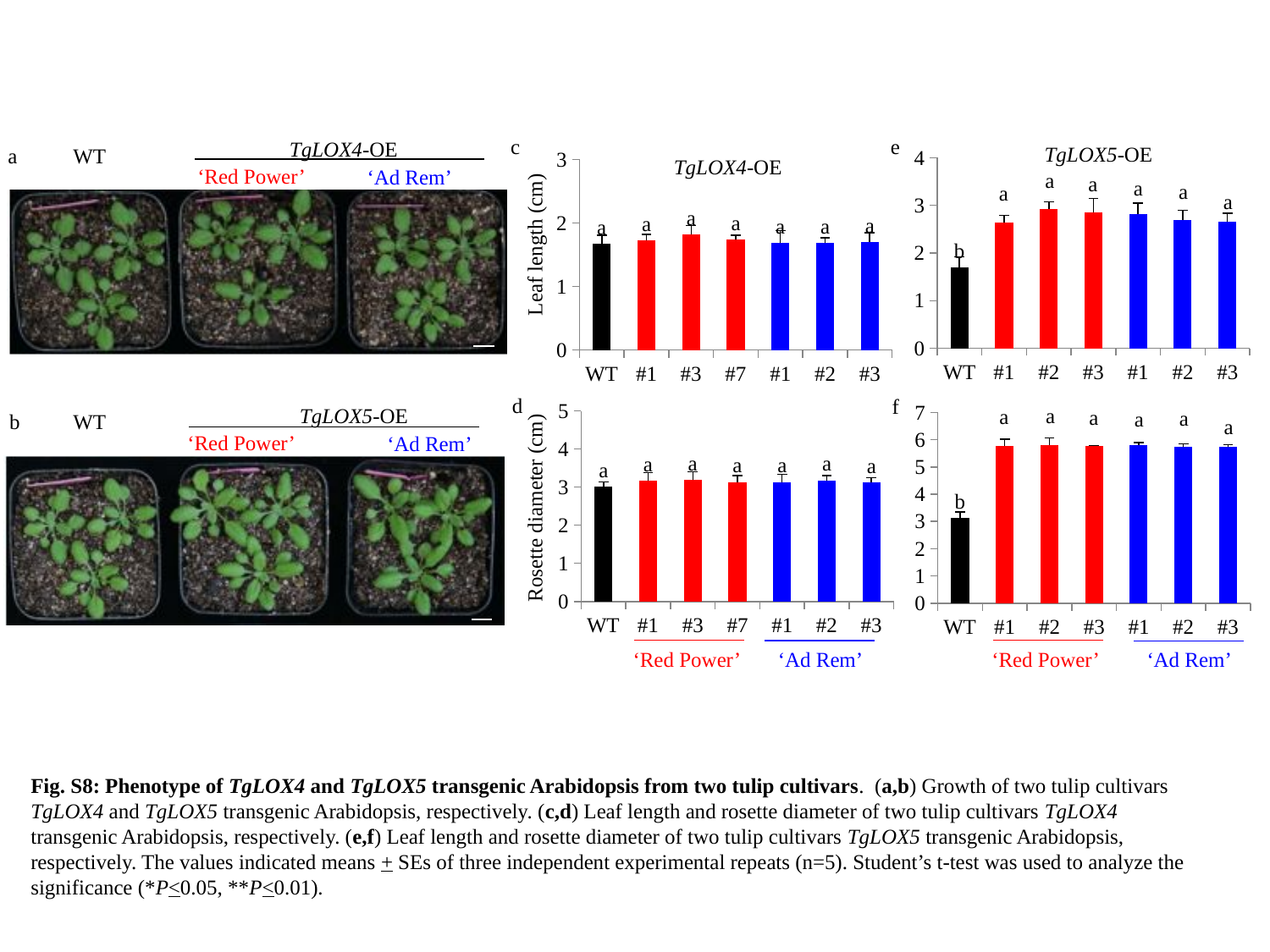

c
e
TgLOX4-OE
TgLOX5-OE
WT
a
### Chart
| Category | |
|---|---|
| WT | 1.7062500000000005 |
| #1 | 2.64 |
| #2 | 2.9200000000000004 |
| #3 | 2.85 |
| #1 | 2.825 |
| #2 | 2.7 |
| #3 | 2.65 |TgLOX4-OE
### Chart
| Category | |
|---|---|
| WT | 1.6736842105263159 |
| #1 | 1.725 |
| #3 | 1.8200000000000003 |
| #7 | 1.7399999999999998 |
| #1 | 1.6900000000000002 |
| #2 | 1.6850000000000005 |
| #3 | 1.705 |‘Red Power’
‘Ad Rem’
Leaf length (cm)
d
f
TgLOX5-OE
### Chart
| Category | |
|---|---|
| WT | 3.015 |
| #1 | 3.16 |
| #3 | 3.185 |
| #7 | 3.135 |
| #1 | 3.135 |
| #2 | 3.18 |
| #3 | 3.125 |
### Chart
| Category | |
|---|---|
| WT | 3.1450000000000005 |
| #1 | 5.775 |
| #2 | 5.815 |
| #3 | 5.76 |
| #1 | 5.805 |
| #2 | 5.74 |
| #3 | 5.74 |b
WT
‘Red Power’
‘Ad Rem’
Rosette diameter (cm)
‘Red Power’
‘Ad Rem’
‘Red Power’
‘Ad Rem’
Fig. S8: Phenotype of TgLOX4 and TgLOX5 transgenic Arabidopsis from two tulip cultivars. (a,b) Growth of two tulip cultivars TgLOX4 and TgLOX5 transgenic Arabidopsis, respectively. (c,d) Leaf length and rosette diameter of two tulip cultivars TgLOX4 transgenic Arabidopsis, respectively. (e,f) Leaf length and rosette diameter of two tulip cultivars TgLOX5 transgenic Arabidopsis, respectively. The values indicated means + SEs of three independent experimental repeats (n=5). Student’s t-test was used to analyze the significance (*P<0.05, **P<0.01).

## Slide 9
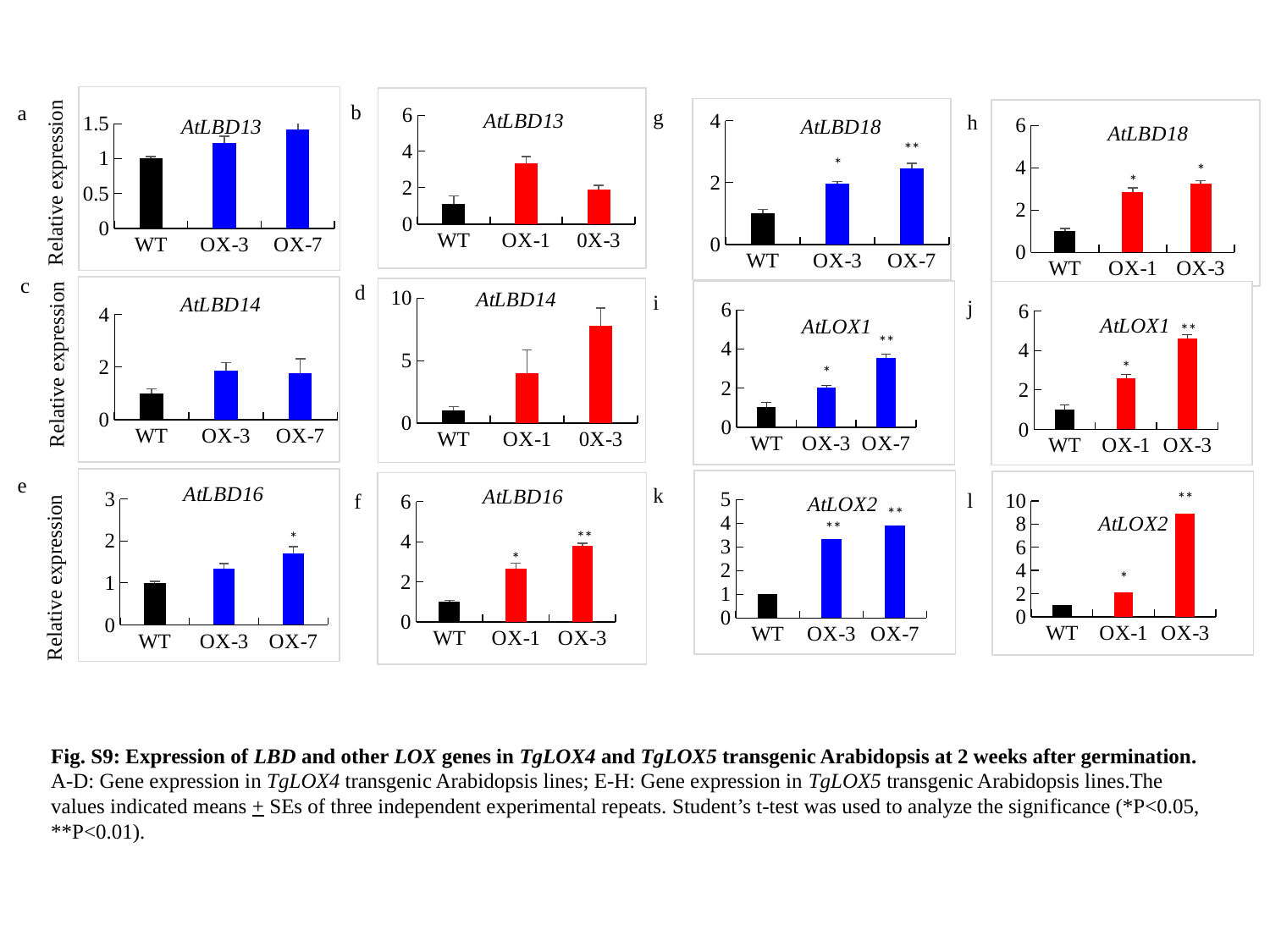

### Chart: AtLBD13
| Category | |
|---|---|
| WT | 1.00033024048937 |
| OX-3 | 1.21689172452435 |
| OX-7 | 1.41186483875105 |
### Chart: AtLBD13
| Category | |
|---|---|
| WT | 1.08303891304561 |
| OX-1 | 3.36582434722876 |
| 0X-3 | 1.88924256650225 |b
a
g
### Chart: AtLBD18
| Category | |
|---|---|
| WT | 1.00302040768018 |
| OX-3 | 1.96029857568345 |
| OX-7 | 2.47026819270588 |
### Chart: AtLBD18
| Category | |
|---|---|
| WT | 1.00428416321955 |
| OX-1 | 2.83627622562843 |
| OX-3 | 3.2501839896449 |h
Relative expression
c
d
### Chart: AtLBD14
| Category | |
|---|---|
| WT | 1.00822632667547 |
| OX-3 | 1.87220730582478 |
| OX-7 | 1.7560243452317 |
### Chart: AtLBD14
| Category | |
|---|---|
| WT | 1.03202345122075 |
| OX-1 | 4.03138077613437 |
| 0X-3 | 7.76764913866086 |
### Chart: AtLOX1
| Category | |
|---|---|
| WT | 1.0202906889051715 |
| OX-3 | 2.0263459439147984 |
| OX-7 | 3.543726028344222 |
### Chart: AtLOX1
| Category | RQ |
|---|---|
| WT | 1.0202857211636127 |
| OX-1 | 2.5860921218725594 |
| OX-3 | 4.612186823120099 |i
j
Relative expression
e
### Chart: AtLBD16
| Category | |
|---|---|
| WT | 1.00044828252966 |
| OX-3 | 1.33518222279959 |
| OX-7 | 1.70885634769444 |
### Chart: AtLOX2
| Category | |
|---|---|
| WT | 1.0022776141580827 |
| OX-3 | 3.346773941179824 |
| OX-7 | 3.8918194606820307 |
### Chart: AtLOX2
| Category | RQ |
|---|---|
| WT | 1.0172871640272032 |
| OX-1 | 2.0692219644356524 |
| OX-3 | 8.924363238835761 |
### Chart: AtLBD16
| Category | |
|---|---|
| WT | 1.00123975191594 |
| OX-1 | 2.65710898993731 |
| OX-3 | 3.80698111151912 |k
l
f
Relative expression
Fig. S9: Expression of LBD and other LOX genes in TgLOX4 and TgLOX5 transgenic Arabidopsis at 2 weeks after germination. A-D: Gene expression in TgLOX4 transgenic Arabidopsis lines; E-H: Gene expression in TgLOX5 transgenic Arabidopsis lines.The values indicated means + SEs of three independent experimental repeats. Student’s t-test was used to analyze the significance (*P<0.05, **P<0.01).

## Slide 10
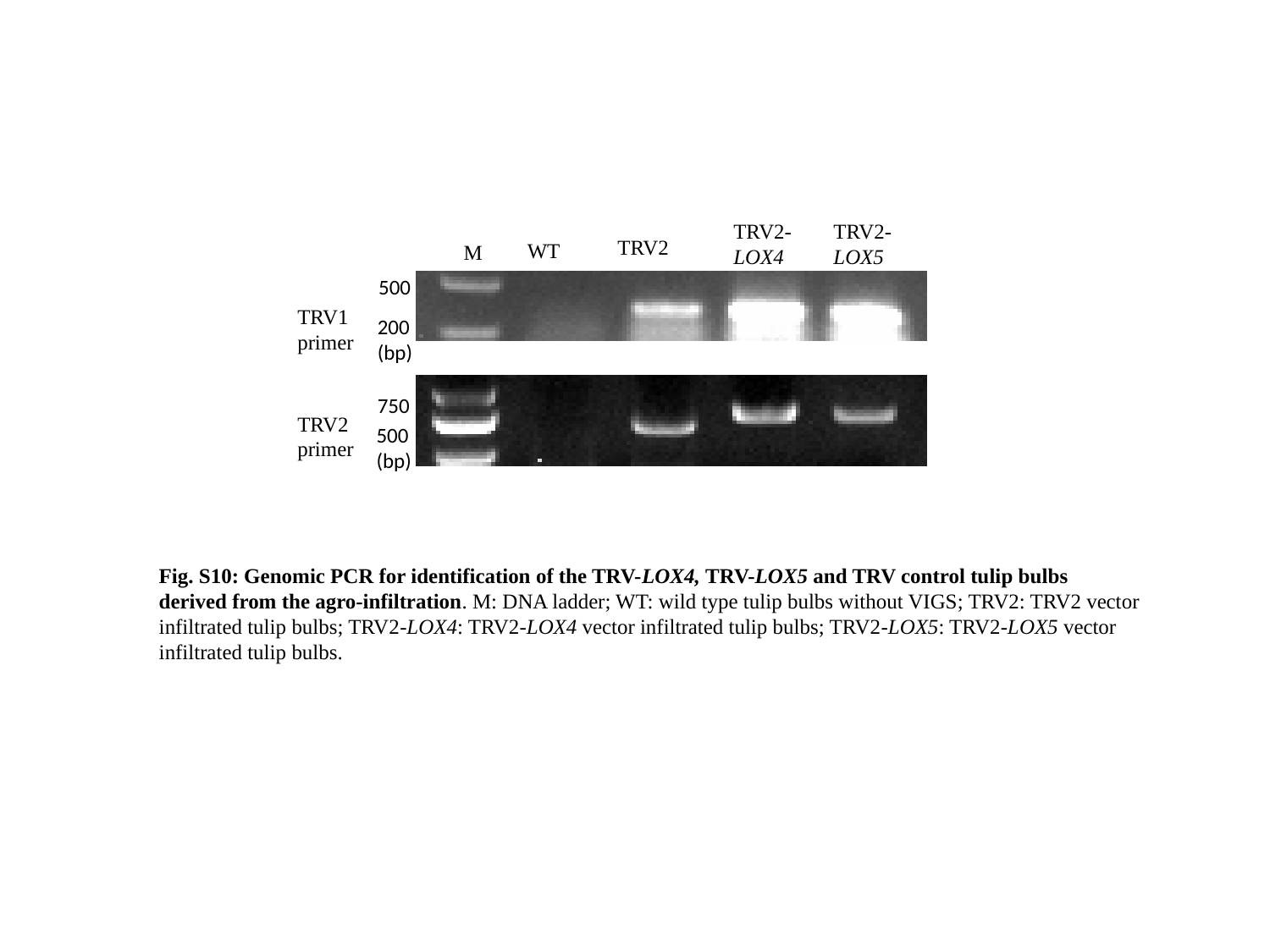

TRV2-LOX4
TRV2-LOX5
TRV2
WT
M
500
TRV1 primer
200
(bp)
750
TRV2 primer
500
(bp)
Fig. S10: Genomic PCR for identification of the TRV-LOX4, TRV-LOX5 and TRV control tulip bulbs derived from the agro-infiltration. M: DNA ladder; WT: wild type tulip bulbs without VIGS; TRV2: TRV2 vector infiltrated tulip bulbs; TRV2-LOX4: TRV2-LOX4 vector infiltrated tulip bulbs; TRV2-LOX5: TRV2-LOX5 vector infiltrated tulip bulbs.

## Slide 11
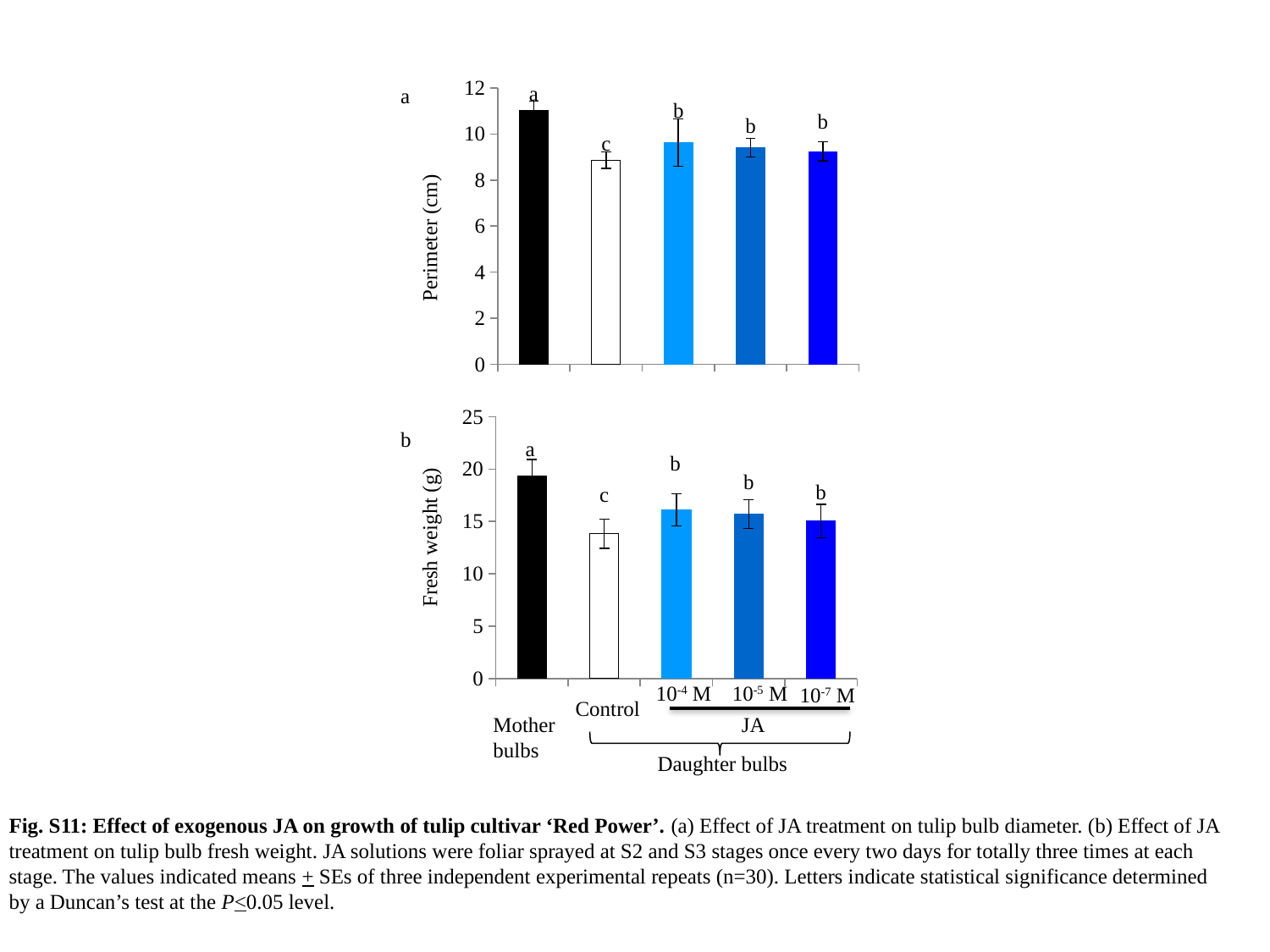

### Chart
| Category | |
|---|---|
| 原始母球 | 11.026 |
| Control | 8.86538461538462 |
| JA-4 | 9.628 |
| JA-5 | 9.408 |
| JA-7 | 9.258 |a
Perimeter (cm)
### Chart
| Category | |
|---|---|
| 原始母球 | 19.3467754 |
| Control | 13.8255769230769 |
| JA-4 | 16.1092 |
| JA-5 | 15.7056 |
| JA-7 | 15.0464 |b
Fresh weight (g)
10-5 M
10-4 M
10-7 M
Control
Mother
bulbs
JA
Daughter bulbs
Fig. S11: Effect of exogenous JA on growth of tulip cultivar ‘Red Power’. (a) Effect of JA treatment on tulip bulb diameter. (b) Effect of JA treatment on tulip bulb fresh weight. JA solutions were foliar sprayed at S2 and S3 stages once every two days for totally three times at each stage. The values indicated means + SEs of three independent experimental repeats (n=30). Letters indicate statistical significance determined by a Duncan’s test at the P<0.05 level.
